# *Natronoglomus mannanivorans* gen. nov., sp. nov., beta-1,4-mannan utilizing natronoarchaea from hypersaline soda lakes

**DOI:** 10.3389/fmicb.2024.1364606

**Published:** 2024-03-12

**Authors:** Dimitry Y. Sorokin, Alexander G. Elcheninov, Nicole J. Bale, Jaap S. Sinninghe Damsté, Ilya V. Kublanov

**Affiliations:** ^1^Winogradsky Institute of Microbiology, Research Centre of Biotechnology, Russian Academy of Sciences, Moscow, Russia; ^2^Department of Biotechnology, Delft University of Technology, Delft, Netherlands; ^3^NIOZ Royal Netherlands Institute for Sea Research, Texel, Netherlands

**Keywords:** hypersaline lakes, haloarchaea, glucomannan, galactomannan, beta-1,4-mannan

## Abstract

Beta-mannans are insoluble plant polysaccharides with beta-1,4-linked mannose as the backbone. We used three forms of this polysaccharide, namely, pure mannan, glucomannan, and galactomannan, to enrich haloarchaea, which have the ability to utilize mannans for growth. Four mannan-utilizing strains obtained in pure cultures were closely related to each other on the level of the same species. Furthermore, another strain selected from the same habitats with a soluble beta-1,4-glucan (xyloglucan) was also able to grow with mannan. The phylogenomic analysis placed the isolates into a separate lineage of the new genus level within the family *Natrialbaceae* of the class *Halobacteria*. The strains are moderate alkaliphiles, extremely halophilic, and aerobic saccharolytics. In addition to the three beta-mannan forms, they can also grow with cellulose, xylan, and xyloglucan. Functional genome analysis of two representative strains demonstrated the presence of several genes coding for extracellular endo-beta-1,4-mannanase from the GH5_7 and 5_8 subfamilies and the GH26 family of glycosyl hydrolases. Furthermore, a large spectrum of genes encoding other glycoside hydrolases that were potentially involved in the hydrolysis of cellulose and xylan were also identified in the genomes. A comparative genomics analysis also showed the presence of similar endo-beta-1,4-mannanase homologs in the cellulotrophic genera *Natronobiforma* and *Halococcoides*. Based on the unique physiological properties and the results of phylogenomic analysis, the novel mannan-utilizing halolarchaea are proposed to be classified into a new genus and species *Natronoglomus mannanivorans* gen. nov., sp. nov. with the type strain AArc-m2/3/4 (=JCM 34861=UQM 41565).

## Introduction

Recent extensive studies on the functional diversity of extremely halophilic archaea belonging to the class *Halobacteria* resulted in a significant shift in a longstanding perception of this unique group of extremophiles as copiotrophic dissipotrophs utilizing either rich amino acid mixtures or simple soluble monomeric compounds, such as sugars or organic acids. In particular, it is becoming evidently clear that many haloarchaeal species possess extensive hydrolytic potential against various groups of recalcitrant insoluble polysaccharides, such as chitin, cellulose, and a range of other neutral glucans (Sorokin et al., 2015, 2018, 2019a,b, 2022a, 2023). In our previous study, we used selective enrichments with a range of soluble and insoluble glucans to obtain pure cultures of haloarchaea utilizing various glucans with different monomers and back-bond structures as growth substrates (Sorokin et al., 2022a). One of the selected groups included five isolates of alkaliphilic haloarchaea that have the ability to grow with beta-mannans, including pure beta-1,4-mannan and its two modifications, glucomannan (with glucose and mannose in the backbone) and galactomannan (with galactose in the side chains). To date, the beta-mannans with backbone have been proven to serve as a growth substrate for three characterized species of haloarchaea: *Natronoarchaeum mannanilyticum* (Shimane et al., 2010) and *Haloarcula mannanilytica* (Enomoto et al., 2020) use galactomannan as a substrate and cellulotrophic *Natronobiforma cellulositropha* uses pure beta-1,4-mannan as a substrate (Sorokin et al., 2018). The latter and the heteropolymeric glucomannan have never been tested before to enrich mannan-utilizing haloarchaea. According to the CAZy database (http://www.cazy.org), the only enzymatically characterized mannan-specific glycoside hydrolases (several subfamilies of the GH family 5 and the GH family 26) are known in bacteria and eukarya, mostly in fungi. This finding certainly indicates a substantial gap in the knowledge on the potential presence of functionally active archaeal beta-mannanases and on the identity of archaea that are able to utilize such polymers as growth substrates.

In this study, we provide taxonomic evaluation and functional genome analysis focusing on glycosyl hydrolase families for several strains of haloarchaea, which were previously enriched from neutral and alkaline hypersaline lakes in southwestern Siberia on insoluble beta-1,4-mannans. All mannan-utilizing isolates are closely related, forming a separate lineage within the family *Natrialbaceae*, and are proposed to be classified as *Natronoglomus mannanivorans* gen. nov., sp. nov.

## Materials and methods

### Media and cultivation conditions

Mix surface sediments and brines collected from several hypersaline salt and soda lakes in the Kulunda Steppe (Altai, Russia) were used as inoculum for enrichment cultures. The brine pH (measured with the field pH meter, WWR) in neutral lakes of the chloride-sulfate type ranged from 7.5 to 8.2, and in soda lakes, the pH values ranged from 9.8 to 11. The total salt concentration (measured in the field with a refractometer and verified by the gravimetry in the laboratory) was found to be ranging from 200 to 400 g l^−1^. Before cultivation, the sediment suspension (one part of sediment:nine parts of brines, v:v) was preincubated for 3 days at 28°C on a rotary shaker with the addition of 200 mg l^−1^ each of ampicillin and streptomycin to suppress the growth of bacteria. The suspended solids were precipitated by centrifugation and resuspended into two types of base mineral medium containing either 4 M total NaCl at pH 7 or a 3:1 (v:v) mix of 4 M NaCl and sodium-carbonate base with 4 M total Na^+^ (the final pH was 9.5). The cultures were supplemented with 1 g l^−1^ each of three types of beta-1,4-mannan (Megazyme, Ireland): pure mannan; glucomannan (beta-1,4 heteropolymer of mannose and glucose with a Gl:Man ratio of 4:1); and galactomannan (beta-1,4-mannan with a side chain decoration of alpha-1,6-linked galactose). The polymers were prepared as 10% suspensions in sterile distilled water and sterilized at 110°C for 30 min, and the enrichments were incubated on a rotary shaker in closed bottles at 37°C until visible substrate degradation (accessed by light microscopy) and the appearance of pink turbidity in the liquid phase occurs. Pure culture isolation was achieved after the plating of final positive serial dilution from single colonies forming the clearance zones on the mannan plates (the mannan suspension was sonicated to reduce the particle size) and showing stable growth in the liquid medium with the beta-1,4-mannan as the sole carbon and energy source.

### Phenotypic characterization

Phase contrast microscopy was performed using Zeiss Axioplane Imaging 2 (Germany). The growth in liquid cultures was monitored OD_600_ after 30 min of gravity sedimentation of insoluble particles or directly in the case of soluble substrates. Thin-section electron microscopy was performed for the cells of type strain AArc-m2/3/4 grown either with beta-mannan or xylan, using a JEOL100 instrument (Japan) as described previously (Sorokin et al., 2018). The ability for anaerobic growth was tested in serum bottles sealed with butyl rubber stoppers after the removal of dissolved oxygen and subjected first to “cold boiling” under a vacuum followed by flashing three times with sterile argon gas. Aerobic substrate utilization, pH-salt profiling, and other standard phenotypic testing were performed as described by Sorokin et al. (2022a). Membrane polar lipids and respiratory menaquinones were extracted from freeze-dried biomass of strains AArc-m2/3/4 and AArc-xg1-1 that were grown with cellobiose at 37°C, 4 M total Na^+^, and pH 9.5 until the late exponential growth phase and were resolved by Ultra High Pressure Liquid Chromatography-High Resolution Mass Spectrometry (UHPLCHRMS) using an Agilent 1290 Infinity I UHPLC (ThermoFisher Scientific), as described previously (Bale et al., 2021; Sorokin et al., 2022b).

Growth tests were performed in a basal alkaline medium containing 4 M total Na^+^ (1 M as sodium carbonates and 3 M as NaCl) at pH 9.5. Substrates were added at a final concentration of 1 g l^−1^ after sterilization from 10% concentrated stocks. In total, 10 ml cultures in 30 ml bottles with screw-capped rubber septum (to prevent evaporation) were incubated on a rotary shaker at 37°C. The growth was monitored by measuring OD_600_ against a control without substrate, directly in the case of soluble substrate, and after vigorous homogenization and 10 min stasis to allow particles to sediment in the case of insoluble polysaccharides.

### Genome sequencing and phylogenomic and functional genomic analyses

The genomes of AArc-m2/3/4 and AArc-xg1-1 were sequenced using the MiSeq Illumina platform and assembled as described previously (Sorokin et al., 2022a). Their draft assemblies were deposited in the GenBank under the numbers GCA_025517485 and GCA_025517495, respectively.

For phylogenomic analysis, 122 conserved single copy archaeal protein markers (Rinke et al., 2021) from the *in silico* translated genomes of two mannan-utilizing natronoarchaea, the closest related strains KZCA124 and TS33, and all described species of the family *Natrialbaceae*, were identified and aligned using the GTDB-tk v.1.7.0 with reference data v.202 (Chaumeil et al., 2019). The resulting alignment was treated using trimAL v1.4.1 with –gt 1 option for complete gap elimination (Capella-Gutiérrez et al., 2009). The phylogenomic tree was constructed and drawn using the RAxML v.8.2.12 (the PROTGAMMAILG model, 1,000 rapid bootstrap replications) and iTOL v.6.5.2, respectively (Stamatakis, 2014; Letunic and Bork, 2019). The whole genome comparisons (ANI, AAI, and POPC) were calculated as described earlier (Sorokin et al., 2023).

Functional genome analysis of the glycoside hydrolases (GH) with particular focus on the mannan-hydrolyzing families including the GH5_7, GH5_8, and GH26 families in AArc-m2/3/4 and AArc-xg1-1 (as well as the genomes of related strains KZCA124 and TS33) was performed using the dbCAN v.4 (Zhang et al., 2018) with HMMER (Mistry et al., 2013) and Diamond tools (Buchfink et al., 2015) to detect the target GH domains. Additional domains were searched using the InterPro database (Paysan-Lafosse et al., 2023). Gene clusters of AArc-m2/3/4, AArc-xg1-1, and several other species containing genes encoding endo-beta-mannanases were visualized using the gggenes package in R (https://cran.r-project.org/web/packages/gggenes). The GH5 and GH26 enzymes of strains AArc-m2/3/4 and AArc-xg1-1 and related proteins found by the NCBI BLAST (the organism list was limited to *Archaea*; the e-value threshold were 1e-10 and 1e-5 for GH5 and GH26, respectively) were used for the phylogenetic reconstruction of the haloarchaeal beta-mannanases. Furthermore, all proteins were analyzed using dbCAN v.4 with HMMER tool to detect target domains; only proteins with target catalytic domains (minimal coverage is 0.4) and some additional carbohydrate-binding domains (minimal coverage is 0.5) were kept in the final protein set. Both sets of sequences (the GH5 and GH26 families) were aligned using MAFFT v.7 with the E-INS-i method (Katoh et al., 2017). The alignments obtained were trimmed using trimAL v1.4.1 with -gt 0.75 (Capella-Gutiérrez et al., 2009). The phylogenetic trees for GH5 and GH26 glycosidases were constructed and decorated as described above for the “ar122”-based phylogenomic tree with a decreased number of bootstrap replications (500).

## Results and discussion

### Enrichment and isolation of pure cultures of mannan-utilizing haloarchaea

Five enrichment cultures (one from neutral salt lakes and four from soda lakes) showed stable growth with four different polymers. The neutral and soda lake enrichments with beta-1,4-mannan yielded strains HArc-m1 and AArc-m2/3/4^T^; the soda lake enrichments with galactomannan yielded strain AArc-glctm5 and with glucomannan yielded strain AArc-gm3/4/5-2. An additional enrichment with xyloglucan provided an additional beta-1,4-mannan-utilizing isolate AArc-xg1-1. It was confirmed that the first four isolates selected with different mannan variations could grow with beta-1,4-mannan. The xyloglucan-yielded isolate AArc-xg1-1 was first identified as a potential mannan utilizer based on its phylogenomic proximity to the mannan-enriched strains and functional genome analysis, and it was later confirmed by a growth test. An interesting fact to note is that the neutral salt lake isolate, HArc-m1, turned out to be identical to its counterpart mannan-selected soda lake natronarchaeon, AArc-m2/3/4, and was confirmed to be able to grow optimally at moderately alkaline conditions used for the enrichment of the soda lake isolates, which is a rare example (at least in our experience). Evidently, two factors might have played a role in such a coincidence: the first is the close proximity of the chloride-sulfate and soda lakes in the southern Kulunda steppe (a few kilometers range) and the second is the unique substrate specialization overruling the significance of the alkali-related adaptation.

### Phenotypic properties

**Figure 1:**
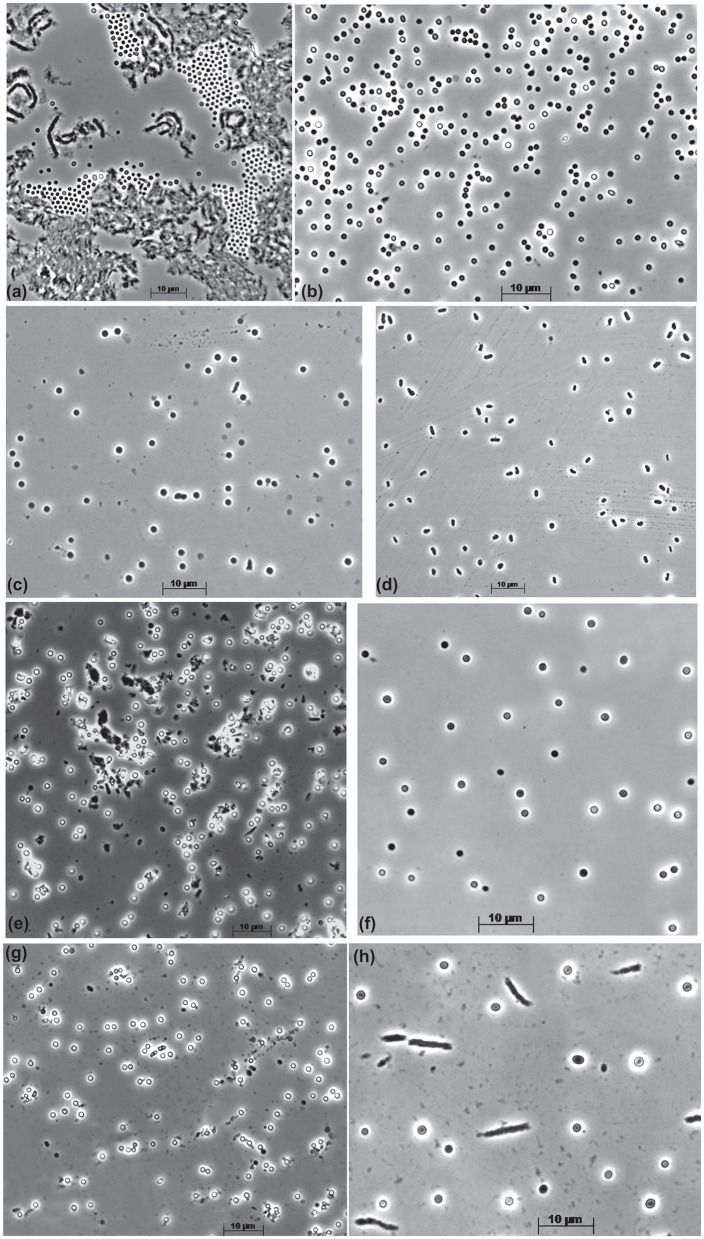
Cell morphology of the beta-1,4-mannan-utilizing haloarchaea growing at 4 M Na^+^, pH 9.5, and 37^o^C. **(a, b)** Strain AArc-m2/3/4 on mannan [**(a)** aggregated cells in the solid phase with polymer, **(b)** free cells]; **(c, d)** strain AArc-xg1-1 [**(c)** on mannan, **(d)** on xyloglucan]; **(e)** strain HArc-m1 on mannan; **(f)** strain AArc-glctm5 on mannan; **(g, h)** strain AArc-gm3/4/5-2 [**(g)** on mannan, **(h)** on glucomannan].

**Figure 2:**
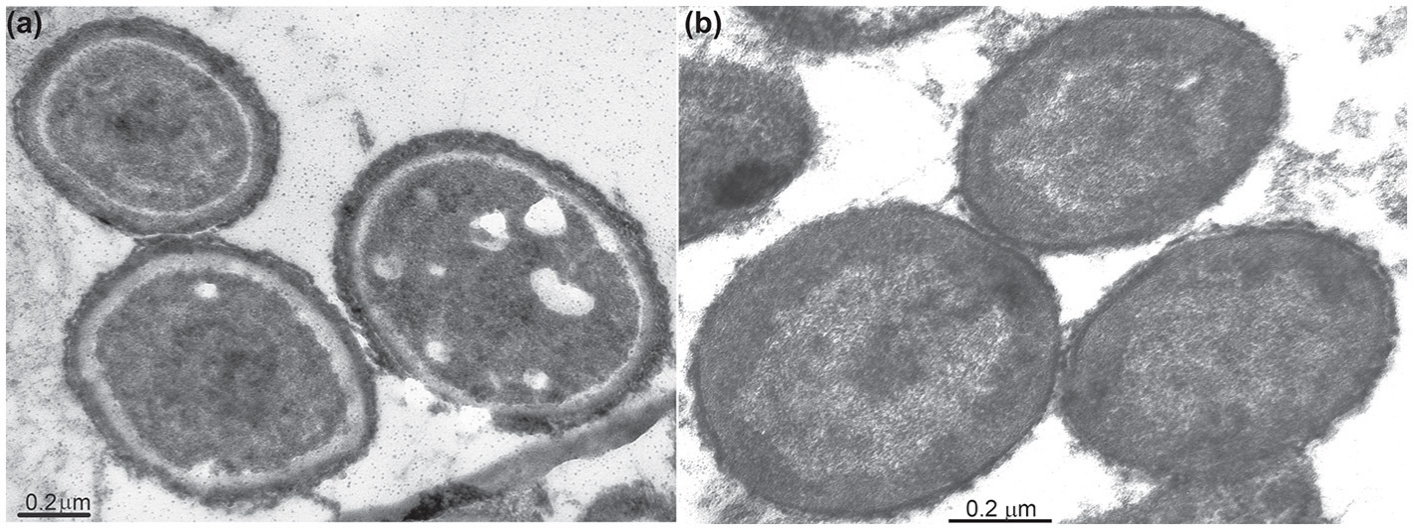
Thin-section electron microscopy microphotographs showing an ultrastructural difference of the AArc-m2/3/4 cells grown with beta-1,4-mannan **(a)** and xylan **(b)**. The mannan-grown cells have an extra external cell layer and extended pseudoperiplasm in comparison to the cells grown on xylan.

The cells of all mannan-utilizing strains were mostly true non-motile cocci that were ~1 μm in diameter, becoming especially refractive when grown with mannan and cellulose (Figure 1). Thin sectioning electron microscopy revealed a major difference in the cell ultrastructure between the mannan- and xylan-grown cells: the former had a much thicker cell wall consisting of several layers and an extended pseudoperiplasmic space (Figure 2), which resembled the cellulose-growing cells of *Natronobiforma* (Sorokin et al., 2018). It might be due to the necessity of these haloarchaea to interact physically with the insoluble glucans.

Membrane phospholipid profiling of strains AArc-m2/3/4 and AArc-xg1-1 indicated domination of C_20_-C_20_ archaeol and C_20_-C_25_ extended archaeol as the core, with phosphatidylglycerolphosphate methyl ester (PGP-Me) and phosphatidylglycerol (PG) as the polar heads in equal proportion (Supplementary Table S1). The glyco- and sulfo-lipids were not detected. The majority of respiratory lipoquinones were represented by the saturated MK-8:8 (75 and 95% in AArc-m2/3/4 and AArc-xg1-1, respectively), and the rest was monounsaturated MK-8:7.

The range of polysaccharides supporting the growth of all isolates included beta-1,4 mannan, glucomannan, amorphous cellulose, xyloglucan, xylan, and arabinoxylan, while galactomannan supported only a weak growth with incomplete degradation. Alpha-glucans from the starch family, beta-glucans with the 1,3- and 1,6-backbone, and the beta-fructans did not support the growth of isolates. Active hydrolysis of the beta-mannan and amorphous cellulose can be observed as the formation of clearance zones around the colonies (Supplementary Figure S1). The range of utilized sugars investigated in the strain AArc-m2/3/4^T^ included galactose, lactose, trehalose, raffinose, rhamnose, sucrose, cellobiose, maltose, melezitose, and melibiose. Interestingly, mannose and glucose, the monomers of two groups of growth-supporting beta-1,4-glucans, were not among the utilized sugars. Most likely, only oligomers were transported inside the cells.

Anaerobic growth with cellobiose as a substrate was not observed both at fermentative conditions and in the presence of various electron acceptors, including nitrate, nitrite, N_2_O, sulfur, thiosulfate, DMSO, and fumarate. Ammonium and urea, but not nitrate, served as the nitrogen source. The cellobiose-dependent growth at pH 9.5 was not inhibited by streptomycin, ampicillin, kanamycin, vancomycin, and tetracycline at concentrations of up to 100 mg l^−1^.

AArc-m2/3/4^T^ grew optimally (with cellobiose at pH 9) at 3.5 M total Na^+^ and with the range from 2.0 to 4.5 M. The pH profiling (with cellobiose at 4 M total Na^+^) was performed for the soda lake isolate, AArc-m2/3/4^T^, and for the salt lake isolate, HArc-m1. The former showed a weak growth starting from pH 7.2 and grew up to pH 9.7, while the latter had a lower growth pH limit starting from a minimum of pH 6.8 and was able to grow up to pH 9.5. Both strains grew optimally around pH 9. Thus, they can be qualified as facultatively alkaliphilic extreme halophiles.

### Phylogenetic analysis

**Figure 3:**
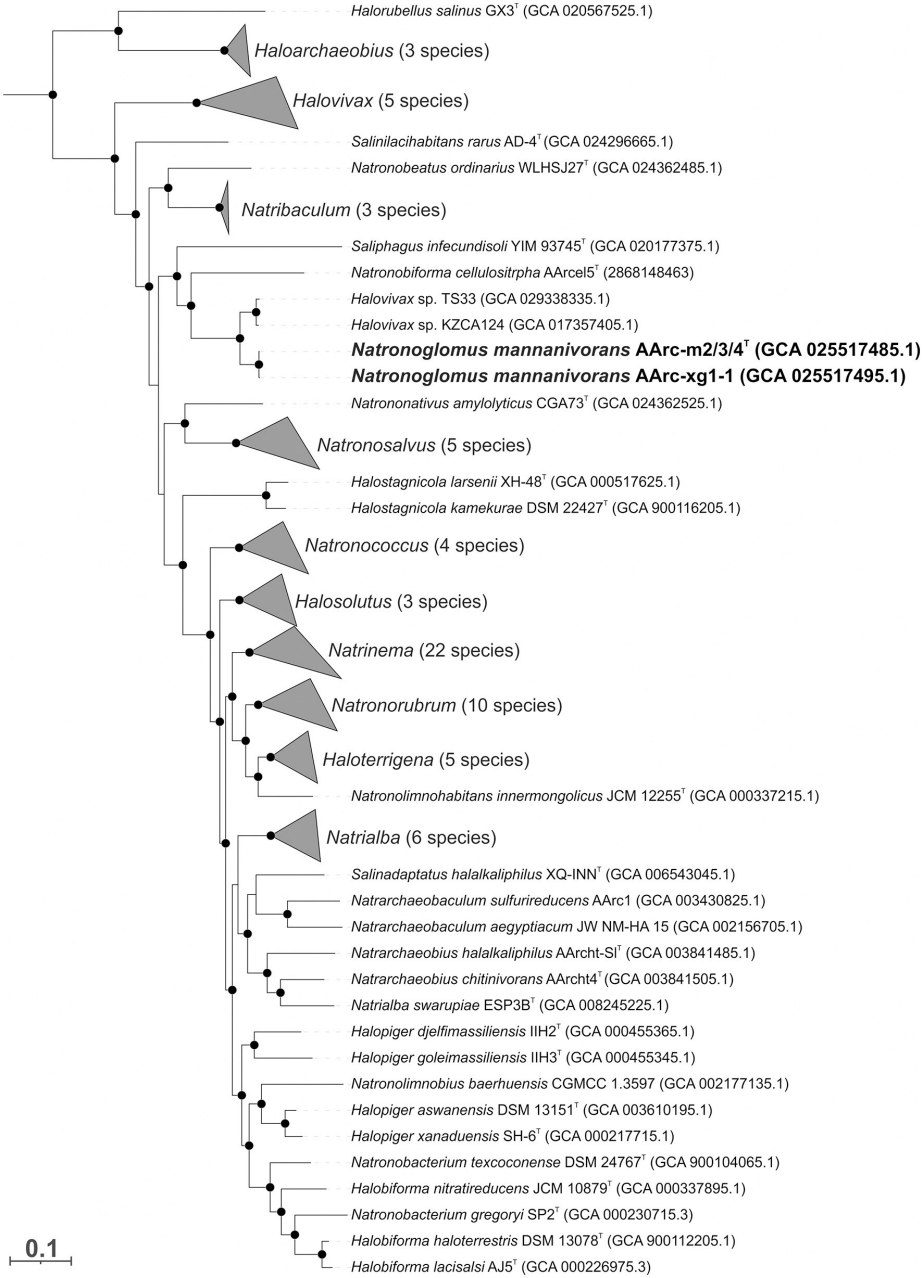
The maximum likelihood phylogenetic tree based on 122 conserved archaeal proteins demonstrating the position of two mannan-utilizing natronoarchaea (in bold) within the family *Natrialbaceae*. The branch lengths correspond to the number of substitutions per site with corrections associated with the models. The black circles at nodes indicate that the percentage of corresponding support values was above 50. The species of some genera are collapsed, and the numbers of species are given in brackets. *Archaeoglobus fulgidus* VC-16^T^, *Methanocella paludicola* SANAE^T^, and *Methanothermobacter thermautotrophicus* Delta H^T^ were used as an outgroup (not shown).

The genomes of AArc-m2/3/4 and AArc-xg1-1 contain a single *rrn* operon with the 16S rRNA gene most closely related to the species of the genera *Halovivax* and *Saliphagus* (~95% sequence identity). However, according to the results of the phylogenomic analysis based on 122 archaeal conserved single-copy protein markers, mannan-utilizing isolates formed a distinct clade within the *Natrialbaceae* family neighboring the cellulotrophic genus *Natronobiforma* (Figure 3, Supplementary Figure S2). Furthermore, the whole-genome-based comparative analyses indicated that the novel isolates form an independent lineage of a new genus level within the *Natrialbaceae* (Supplementary Table S2). Average nucleotide identities (ANI) between the AArc-m2/3/4^T^ and AArc-xg1-1 strains was 98.5%, which means that they belong to the same species; ANI values between novel isolates and the representatives of the closest genera, *Natronobiforma* and *Saliphagus*, were 77.9 and 77.1%, respectively, and the range for other type species of the *Natrialbaceae* varied from 74.1 to 78.6%. Average amino acid identity (AAI) values between the AArc-m2/3/4^T^ and AArc-xg1-1 strains and the type species of the *Natrialbaceae* ranged from 58.9 to 70.3%, with the highest value being *N. cellulositropha*. These values are significantly below the threshold for the demarcation of genera within the *Natrialbaceae* (76%; de la Haba et al., 2021). The percentage of conserved proteins (POCP) between novel strains and the type species of the *Natrialbaceae* varied from 49.4 to 66.2% with the highest values being *N. cellulositropha* and *Saliphagus infecundisoli*. The highest POCP values were slightly higher than the proposed genus-level threshold of 50% (Qin et al., 2014). However, many authors often indicated that a strict boundary of 50% is not suitable for the demarcation of genera within some taxa (Aliyu et al., 2016; Orata et al., 2018; Wirth and Whitman, 2018). Thus, it is likely that, in the case of the *Natrialbaceae*, the POCP threshold needs to be revised.

### Functional genome analysis

#### Beta-mannan utilization potential

The main functional property of the new isolates is the potential to utilize insoluble beta-1,4-mannans and beta-1,4-glucans (cellulose) as a growth substrate. The two sequenced genomes contained a very similar array of genes encoding the key glycoside hydrolases that were potentially involved in the unique substrate specificity of the novel natronoarchaea. A most interesting group of such enzymes included beta-1,4-mannanases belonging to the families GH26 (one enzyme), GH5_7 (four enzymes, two of them with a carbohydrate binding domain, CBM), and GH5_8 (one enzyme with a CBM) (Table 1). All six proteins have TAT translocation signals (i.e., are extracellular) and are co-located in two large genomic islands together with the genes coding for multiple endo-beta-1,4-glucanases from the GH5 superfamily, which is most likely responsible for cellulose degradation and/or endo-beta-1,4-xylanases from the GH10 family.

**Table 1:** Polysaccharide hydrolases potentially involved in the beta-mannan, cellulose, and xylan hydrolysis encoded in the genome of mannan-utilizing strain AArc-m2/3/4.

**Locus tag MCU4972+**	**Protein family**	**Putative function**	**Protein size (aa)**	**Localization (signal peptide)**
0716	GH5	Endo-beta-1,4-glucanase	523	(E) TAT/SPI
2295	GH11	Endo-beta-1,4-xylanase	428	(E) TAT/SPI
2297	GH10/2 × CBM85	Endo-beta-1,4-xylanase	1,035	(E) TAT/SPI-SPII
2716	GH5/PKD	Endo-beta-1,4-glucanase	637	(E) TAT/SPI
2717	GH5	Endo-beta-1,4-glucanase	610	(E) TAT/SPI
2718	GH5/CBM6	Endo-beta-1,4-glucanase	1,755	(E) TAT/SPI
2719	–	S-layer glycoprotein	258	(M) 2 TMH
2720	**GH5_7/CBM5**	**Endo-beta-1,4-mannanase**	759	(E) TAT/SPI
2721	GH5	Endo-beta-1,4-glucanase	646	(E) Sec/SPI
2722	GH10	Endo-beta-1,4-xylanase	589	(E) TAT/SPI
2723	GH10	Endo-beta-1,4-xylanase	741	(E) TAT/SPI
2724	GH10	Endo-beta-1,4-xylanase	577	(E) TAT/SPI
2725	GH5	Endo-beta-1,4-glucanase	600	(E) TAT/SPI
2726	–	Dockerin/PKD family	815	(E) TAT/SPI
2727	CBM6	Cellulose-binding	592	(E) TAT/SPI
2728	GH5	Endo-beta-1,4-glucanase	598	(E) TAT/SPI
2729	GH5/CBM6	Endo-beta-1,4-glucanase	835	(E) TAT/SPI
2730	GH5	Endo-beta-1,4-glucanase	490	(E) TAT/SPI
2732	CBM3	Chitin/cellulose-binding	578	(E) TAT/SPI
2733	**GH5_7**	**Endo-beta-1,4-mannanase**	469	(E) Sec/SPI
2734	**GH5_7**	**Endo-beta-1,4-mannanase**	779	(E) TAT/SPI
2735	GH5/CBM6	Endo-beta-1,4-glucanase	827	(E) TAT/SPI
3710	GH9	cellulase	821	(E) TAT/SPI
4111	GH5/CBM9	Endo-beta-1,4-glucanase	653	(E) TAT/SPI
5514	**GH5_7**	**Endo-beta-1,4-mannanase**	417	(E) TAT/SPI
5518	**GH26**	**Endo-beta-1,4-mannanase**	466	(E) TAT/SPI
5520	**GH5_8**/CBM6	**Endo-beta-1,4-mannanase**	407	(E) TAT/SPI
5524	GH2	Beta-mannosidase	844	(C)

(E), extracellular; (C), cytoplasmic; CBM, carbohydrate-binding domain. Mannan-specific enzymes are in bold.

**Figure 4:**
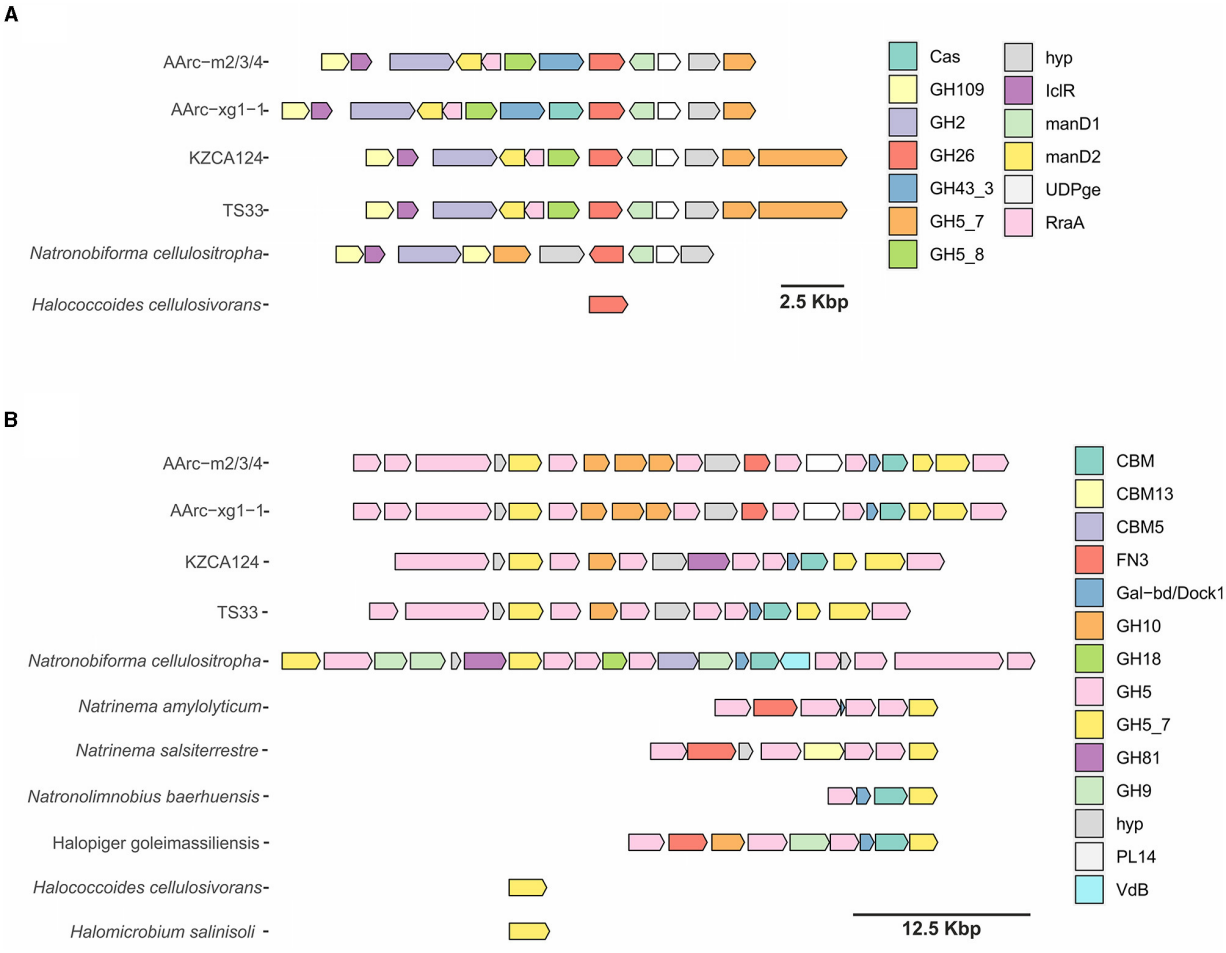
Clusters containing endo-beta-mannanase genes of AArc-m2/3/4^T^ and AArc-xg1-1 strains and similar gene clusters of other haloarchaeae encoding GH26, GH5_7, and GH5_8 families **(A)** or enzymes of the GH5_7 family only **(B)**: Cas, CRISPR-associated nuclease; GHx, glycoside hydrolase of “x” family; hyp, hypothetical protein; IclR, HTH-type transcriptional regulator of IclR family; manD, mannonate dehydratase; UDPge, UDP-glucose 4-epimerase; RraA, putative oxaloacetate decarboxylase of RraA family; CBM, carbohydrate-binding module; FN3, fibronectin type III domain-containing protein; Gal-db/Dock1, galactose-binding-like domain- and/or type I dockerin domain-containing protein; PL14, polysaccharide lyase of PL14 family; and VbB, vanadium-dependent bromoperoxidase.

The first cluster contains genes of three endo-beta-1,4-mannosidases from the GH5_7 and GH5_8 subfamilies and the GH26 family and a beta-mannosidase from the GH2 family (Figure 4A). Moreover, endo-alpha-1,5-L-arabinanase from the GH43 family, a putative enzyme from the GH109 family (homologous to various sugar dehydrogenases), two mannonate dehydratases, putative UDP-glucose 4-epimerase, and a transcriptional regulator of the IclR family were encoded in this cluster. In the genome of AArc-xg1-1, there was an additional CRISPR-associated endonuclease. Very similar clusters were found in the genomes of undescribed strains KZCA124 and TS33, which were closely related to our mannan isolates, but they possessed an additional GH5_7 gene, while genes of GH43 glycosidase were absent. Homologous cluster in *N. cellulositropha* AArcel5 lacked genes of GH5_8 enzyme and one mannonate dehydratase gene, while *Halococcoides cellulosivorans* HArcel1 had only a single endo-beta-1,4-mannosidase from GH26.

The second cluster was much larger and encoded up to 21 proteins (Figure 4B). Identical clusters encoding 11 glycosides from the GH5 family (both endo-beta-1,4-mannanases and endoglucanases), three endo-beta-1,4-xylanases from the GH10 family, putative polysaccharide lyase (PL)14 with an unknown function, and several proteins containing putative CBM domains (galactose-binding-like domain, fibronectin type III, and type I dockerin) were observed in the genomes of AArc-m2/3/4 and AArc-xg1-1. Clusters in the genomes of KZCA124 and TS33 did not encode proteins of the PL14 family and fibronectin type III domain-containing proteins. Moreover, there were fewer genes of endo-glucanases from GH5 and GH10 enzymes. The largest endo-glucanase encoding cluster was found in the genome of *N. cellulositropha* AArcel5 with 10 x GH5 and GH9 endo-beta-1,4-glucanases, GH81 endo-beta-1,3-1,4-glucanase but lack the GH10 xylanases. Other clusters of the second type consisted of four to nine genes (mostly genes of glycosidases from GH5 and GH5_7). Both *H. cellulosivorans* and *Halomicrobium salinisoli* had a single gene encoding endo-beta-1,4-mannanases from the GH5_7 subfamily.

**Figure 5:**
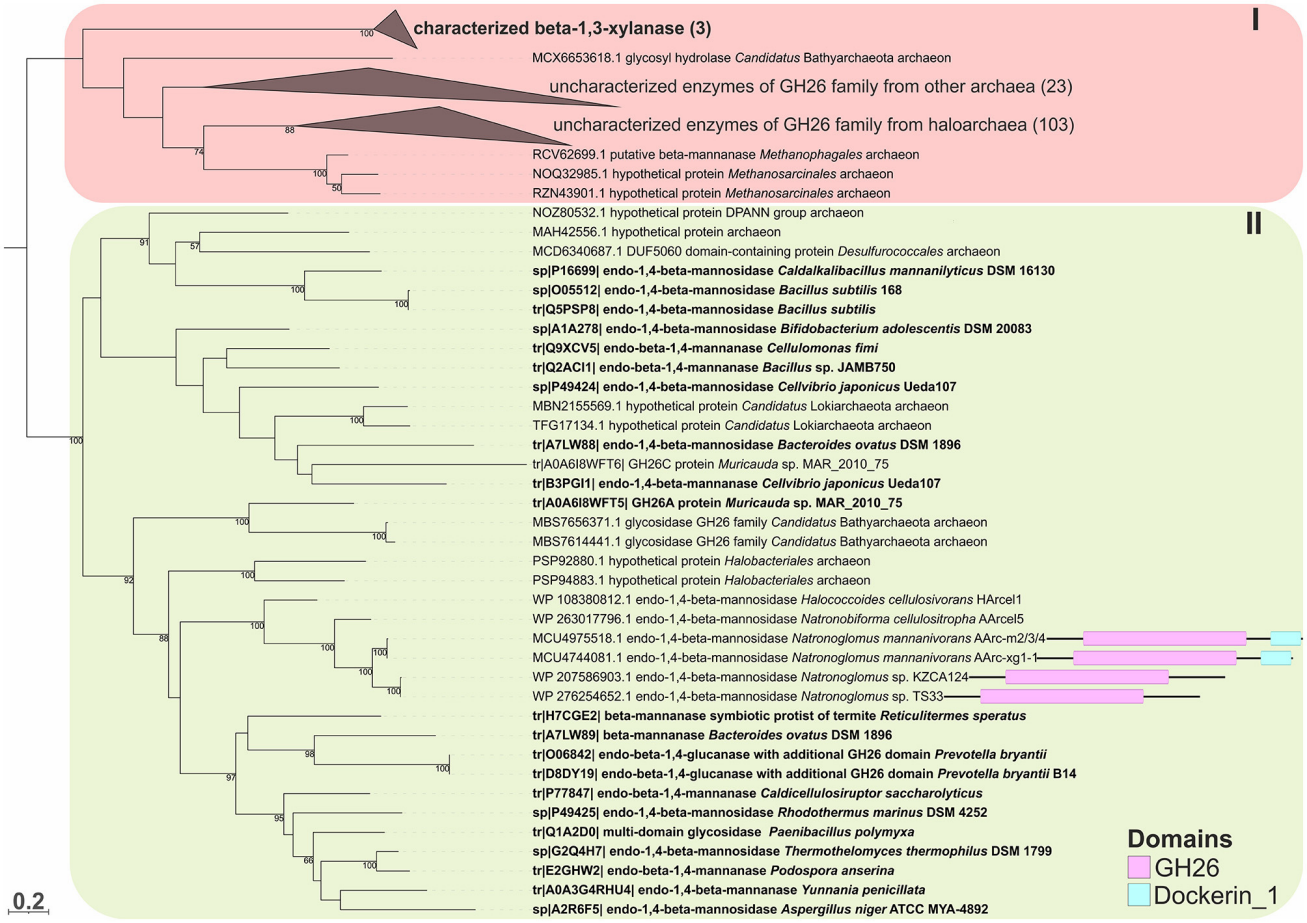
The phylogenetic analysis of the GH26 family endo-beta-1,4-mannanases (with domain architecture) of strains AArc-m2/3/4^T^ and AArc-xg1-1 and closely related strains KZCA124 and TS33. Biochemically characterized enzymes are highlighted in bold. Domain architectures for endo-beta-1,4-mannosidases of AArc-m2/3/4, AArc-xg1-1 strains, and its closest relative are given. The numbers at nodes indicate the percentage of corresponding support values (only values higher than 50% are shown).

**Figure 6:**
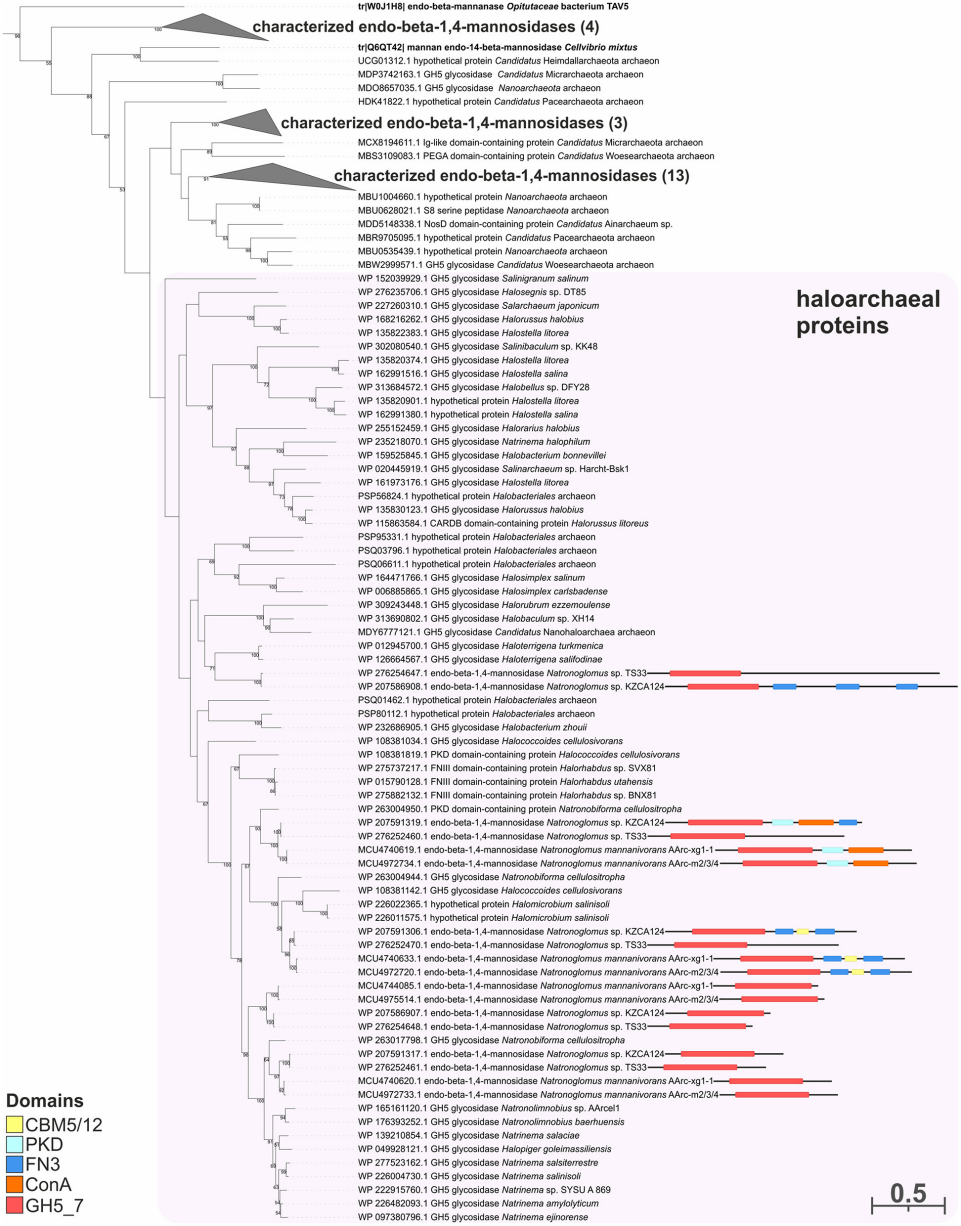
The phylogenetic analysis of enzymes of strains AArc-m2/3/4 and AArc-xg1-1 belonging to the GH5 family (the GH5_7 subfamily). Biochemically characterized enzymes are highlighted in bold. Domain architectures for endo-beta-1,4-mannosidases of AArc-m2/3/4, AArc-xg1-1 strains, and its closest relative (KZCA124 and TS33) are given. The numbers at nodes indicate the percentage of corresponding support values (only values higher than 50% are shown).

**Figure 7:**
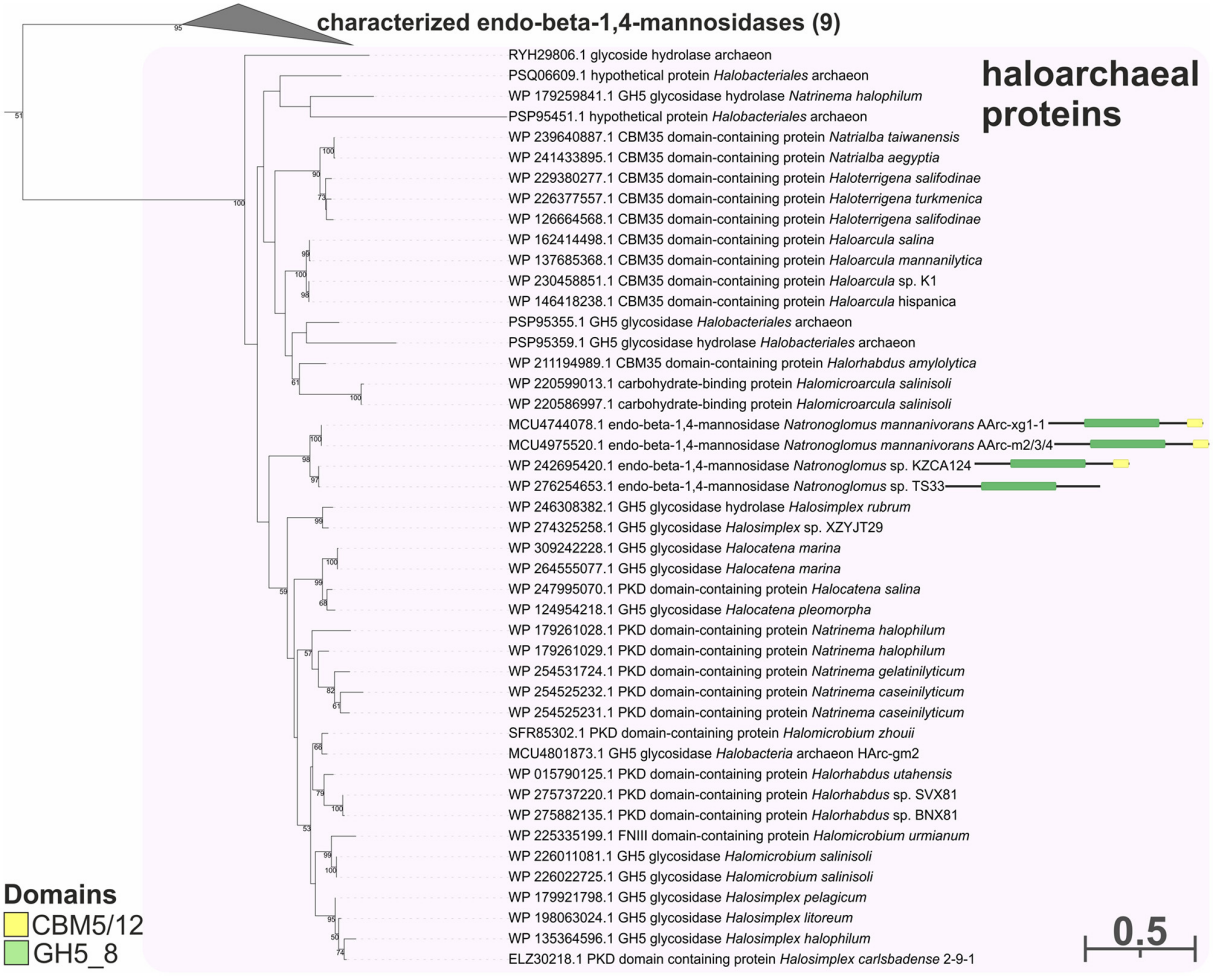
The phylogenetic analysis of enzymes of strains AArc-m2/3/4 and AArc-xg1-1 belonging to the GH5 family (the GH5_8 subfamily). Domain architectures for endo-beta-1,4-mannosidases of AArc-m2/3/4, AArc-xg1-1 strains, and its closest relative (KZCA124 and TS33) are given. The numbers at nodes indicate the percentage of corresponding support values (only values higher than 50% are shown).

To predict the functions of the GH26 and GH5_7/8 subfamilies more reliably, a phylogenetic analysis of these families was performed. This analysis demonstrated that the GH26 encoded in the genomes of the AArc-m2/3/4^T^ and AArc-xg1-1 strains are indeed endo-beta-1,4-mannanases belonging to the clade containing biochemically characterized beta-mannanases from bacteria and fungi (Figure 5). In addition to the catalytic GH26 domain, these enzymes contain the dockerin type I domain participating in the binding of substrate. Enzymes of KZCA124 and TS33, as well as *N. cellulositropha* and *H. cellulosivorans*, also belong to this clade (II). It is remarkable that there were more than 100 proteins of the GH26 family from halophilic archaea (and other archaea) belonging to another clade (I), which have a very low level of homology with the confirmed beta-1,4-mannanases. It is likely that these proteins together with the characterized beta-1,3-xylanases should be separated into a different subfamily of the GH26 or even into a separate family. Due to the complexity of the GH5 family, recently including 57 subfamilies (Supplementary Figure S3), analysis of these glycosidases was focused on clades containing endo-beta-mannosidases (GH5_7 and GH5_8 subfamilies). Both AArc-m2/3/4^T^ and AArc-xg1-1 strains possess four enzymes from the GH5_7 subfamily (Figure 6), of which two of them contained additional domains (carbohydrate-binding module family 5/12, fibronectin type III or PKD, and concanavalin A-like lectin domains), which may enhance the hydrolytic activity against insoluble polysaccharides. In addition to the abovementioned enzymes, KZCA124 and TS33 had another protein of GH5_7 containing three fibronectin type III domains. Moreover, one enzyme of the GH5_8 subfamily was encoded in each genome (Figure 7). These proteins consisted of a catalytic domain and the CBM5/12. Both GH5_7 and GH5_8 enzymes from the studied haloarchaea were clustered together with biochemically characterized endo-beta-1,4-mannonases from bacteria, fungi, and plants indicating that haloarcheal proteins should also possess endo-mannonolytic activity.

#### Other repertoire of polysaccharide hydrolases encoded in the genomes

In addition to the mannanase-cellulase-xylanase GH families, both sequenced genomes also encoded a large repertoire of other polysaccharide-active enzymes including putative PL families listed in Supplementary Tables S3 and S4. The major difference between these groups of hydrolases is cell localization: the former are generally all extracellular, while many proteins from the latter group are cytoplasmic. The putative substrate for such enzymes includes cellulose/xyloglucan (GH3, GH5, and GH9 families), xylan/arabinoxylan (GH3, GH10, GH11, GH51, GH67, and GH115 families) arabinogalactan (GH2, GH30, GH42, GH43, GH51, and GH154 families), starch (GH13 family), acidic polysaccharides, such as polygalacturonan (PL1, PL22, and GH28), rhamnogalacturonan (PL11, PL26, PL42, GH4, and GH105), and alginate, and their oligomers. However, growth and hydrolytic activity were only observed with arabinoxylan and xyloglucan.

#### Central sugar metabolism

**Figure 8:**
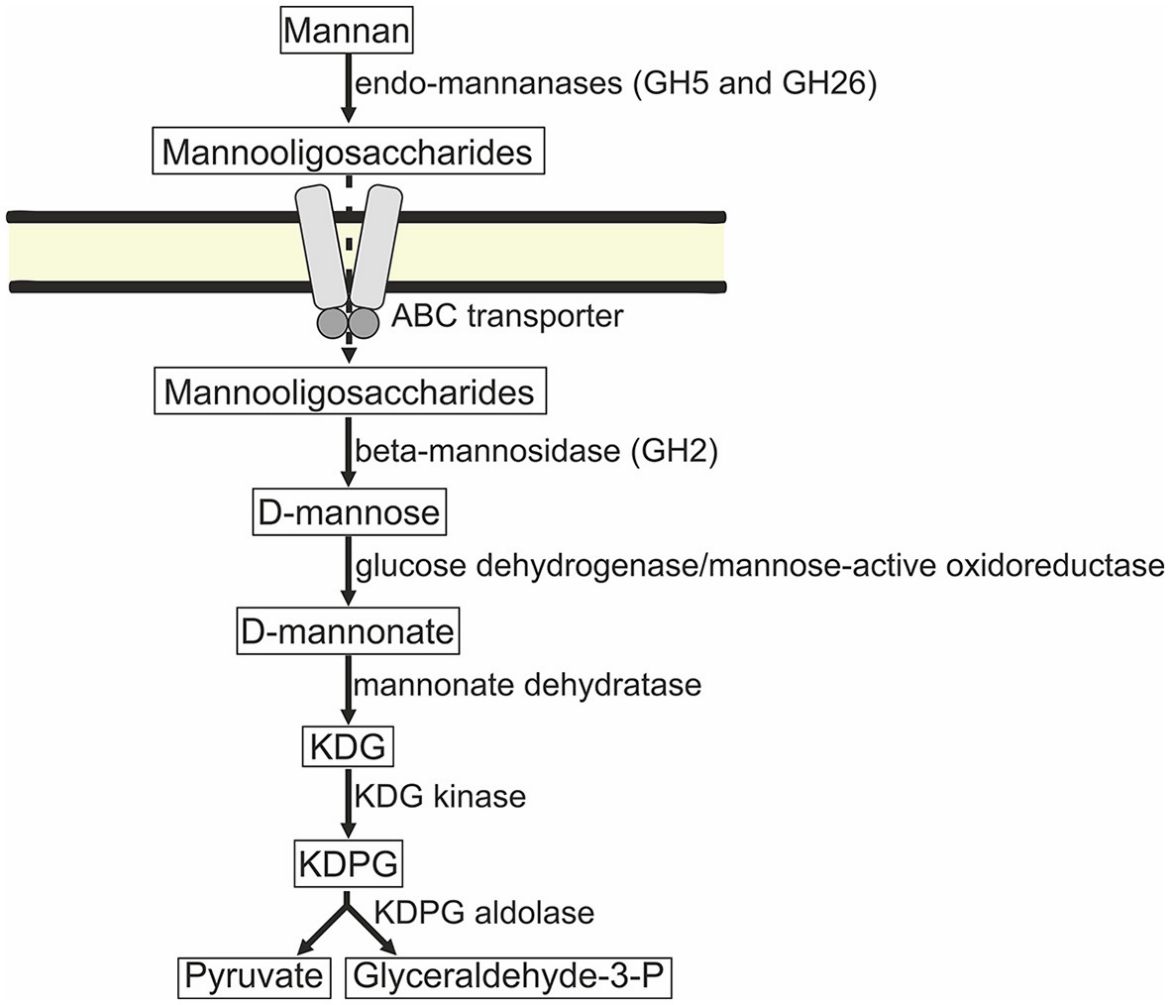
Predicted mannose catabolism pathway in strains AArc-m2/3/4 and AArc-xg1-1. ABC, ATP-binding cassette; KDG, 2-dehydro-3-deoxy-D-gluconate; KDPG, 2-dehydro-deoxy-6-phosphogluconate.

Taking into account that the GH2 family beta-mannosidases are intracellular and the strains are unable to grow on mannose, it is plausible to assume that mannan is hydrolyzed to mannooligosaccharides outside the cells, and then, the oligomers are imported into the cells. The phosphotransferase system (PTS) transporter genes were not found in the genomes of AArc-m2/3/4^T^ and AArc-xg1-1 similar to cellulotrophic haloarchaea (Elcheninov et al., 2023). On the other hand, there were multiple genes of putative carbohydrate-specific ABC transporters (e.g., MCU497+ 1351–1354; 1378–1381; 3422–3426; 4795.1–4797.1; 4083–4081; and 5056–5061) in AArc-m2/3/4^T^, some of which likely import mannooligosaccharides into the cells, and furthermore, they are hydrolyzed by the GH2 beta-mannosidases. It is likely that mannose is oxidized during the side activity of glucose dehydrogenase or putative oxidoreductase (MCU4975802) distantly homologous to mannose-specific aldohexose dehydrogenase from *Thermoplasma acidophilum* (Nishiya et al., 2004). Under the action of mannonate dehydratases ManD (the genes of two of them, 5517 and 5522, were co-located in the gene cluster with mannanases), mannose is converted to 2-dehydro-3-deoxy-D-gluconate. Finally, it enters into the semi-phosphorylative Entner–Doudoroff (ED) pathway with the generation of glyceraldehyde-3-phosphate and pyruvate (Figure 8). Genes encoding enzymes of terminal steps in the ED pathway were also present: 2-dehydro-3-deoxy-D-gluconate kinase (3928 and 5533) and 2-dehydro-deoxy-6-phosphogluconate aldolase (2034).

#### Other metabolically important traits

Other functionally important proteins from the genome of AArc-m2/3/4^T^ are listed in Supplementary Table S5, including the ion-pH homeostasis, energy-related respiratory complexes, and some others. In particular, an incomplete denitrification pathway is encoded, including Cu-nitrite reductase NirK, archaeal type of NO-reductase qNor, and N_2_O reductase, but lacks nitrate reductase Nar. Despite this, the organisms did not grow anaerobically with either nitrite or N_2_O as acceptors (with cellobiose as substrate).

## Taxonomy conclusion

Comparative properties of the novel isolates in relation to the type species of the most related genera are shown in Table 2. The most similar in properties from the three related genera is obviously *N. cellulositropha*, but it was unable to grow with glucomannan and galactomannans, and its sugar utilization spectrum is much more restricted. The other two species of the related genera (*Natronosalvus* and *Saliphagus*) lack the beta-mannanase genes in their genomes and, thus, most likely would be able to grow with this polysaccharide type. In addition, *S. infecundisoli* is a neutraphilic haloarchaeon unable to grow at high pH. Furthermore, the mannan-utilizing natronoarchaea nearly lacked glycolipids, while in *Natronosalvus* and *Saliphagus*, several glycolipids constituted a large fraction of the membrane polar lipids, and the latter also has sulfolipid PGS not usually present in the soda lake natronoarchaea (Bale et al., 2021).

**Table 2:** Comparative properties of the β-mannan utilizing isolates with the closest related genera from the family *Natrialbaceae* (Sorokin et al., 2018; Yin et al., 2018; Tan et al., 2023).

**Property**	***“Natronoglomus mannanivorans*”**	***Natronobiforma cellulositropha***	***Natronosalvus amylolyticus***	***Saliphagus infecundisoli***
The number of isolates	5	4	1	2
Cell morphology	Non-motile cocci	Motile flat rods or non-motile cocci	Non-motile rods or angular coccoids	Non-motile cocci
Pigmentation	Red	Pink	Red	Pink-yellowish
Anaerobic growth	–	–	–	–
**Substrates for aerobic growth**				
Polysaccharides	Beta-mannans, cellulose, xylan, xyloglucan, and arabinoxylan	Cellulose, xylan, and beta-mannan	Starch	Starch
Sugars	Galactose, lactose, rhamnose, raffinose, sucrose, cellobiose, maltose, trehalose, melezitose, and melibiose	Cellobiose and maltose	Mannose, galactose, and sucrose	Glucose, mannose, raffinose, sucrose, maltose, and atrehalose
Others	–	–	Pyruvate, alanine, and ornithine	Pyruvate, succinate, glutamate, aspartate, lysine, and ornithine
Beta-mannanase genes	+ (6)	+(4)	–	–
Amylase	–	–	+	+
Esterase/lipase	– (tributyrin)	– (tributyrin)	– (Tween-80)	+ (Tween-20)
Protease	– (casein)	– (casein)	+ (casein)	+ (casein)
Catalase/oxidase	+/+	+/+	nd	+/+
Indole from tryptophane	+	–	–	–
Salinity range (optimum) M Na^+^	2.0–4.5 (3.5)	2.5–4.8 (4.0)	0.9–4.8 (3.4)	2.0–6.0 (2.5–3.0)
pH range (optimum)	7.2–9.7 (9.0–9.2)	7.5–9.9 (8.5–9.0)	6.0–9.5 (8.0–8.5)	6.5–8.5 (7.0–7.5)
Temperature max (^o^C)	48 (at pH 8.5)	53 (at pH 8.5)	55^*^	50 (at optimum pH)
Core lipids (archaeols)	C_20_-C_20_, C_20_-C_25_ DGE	C_20_-C_20_, C_20_-C_25_ DGE	C_20_-C_20_, C_20_-C_25_ DGE	nd
**Intact polar lipids**				
Phospholipids	PG, PGP-Me	PG, PGP-Me	PG, PGP-Me, PA	PG, PGP-Me, PGS
Glycolipids	MG (trace amount)	MG, DG	DGD-1, S-DGD-1	S-DGD-1
			S-TGD-1	
Respiratory lipoquinones	MK-8:8 (major)	nd	nd	nd
	MK-8:7 (minor)			
	MK-7:7 (trace amount)			
DNA G + C (%, genomic)	62.0 (2 strains)	65.5 (1 strain)	63.7	64.0
Isolation source	Inland hypersaline salt and soda lakes	Inland hypersaline soda lakes	Hypersaline alkaline lake	Saline soil

nd, not reported.

^*^The pH is not reported.

Lipids: PA, phosphatidic acid; PG, phosphatidylglycerol; PGP, phosphatidylglycerophosphate; PGP-Me, phosphatidyl-glycerophosphate methyl ester; PGS, phosphatidylglycerol sulfate; MG, phosphatidyl monoglycosyl diether; DG, phosphatidyl diglycosyl diether; DGD-1, mannosyl glycosyl diether; S-DGD-1, sulfated mannosyl glycosyl diether; S-TGD-1, sulfated galactosyl mannosyl glycosyl diether; and DGE, dialkyl glycerol ether.

In conclusion, taking into account the unique functional specialization and distant phylogeny, the beta-mannan-utilizing natronoarchaea from Siberian hypersaline soda lakes are proposed to form a new genus and species *Natronoglomus mannanivorans*.

### Description of *Natronoglomus mannanivorans* gen. nov., sp. nov.

#### *Natronoglomus* gen. nov.

Na.tro.no.glo'mus. Gr. neut. n. *natron* arbitrarily derived from Arabic n. *natrun* or *natron*, soda; L. neut. n. *glomus*, a ball; N.L. neut. n. *Natronoglomus*, a coccoid natronoarchaeon.

Natrononoarchaea from hypersaline lakes with mostly coccoid cells. Aerobic organoheterotrophs with the ability to utilize insoluble beta-mannans and cellulose as growth substrates. Extremely halophilic and moderately alkaliphilic. The dominant polar membrane lipids are phosphatidylglycerophosphate methyl ether (PGP-Me) and phosphatidylglycerol (PG) with C_20_-C_20_ and C_20_-C_25_ archaeol cores. The genus belongs to the *Natrialbaceae* family, class *Halobacteria*. A three-letter abbreviation is *Ngm*.

#### *Natronoglomus mannanivorans* sp. nov.

man.na.ni.vo'rans. N.L. neut. n. *mannanum*, mannan; L. pres. part. *vorans*, devouring; N.L. part. adj. *mannanivorans*, mannan devouring.

The cells are mostly non-motile cocci of 0.8–1.2 μm producing red pigments. The cells grown on mannan have a thick cell wall and extended pseudoperiplasm. The cells lyze in distilled water. The colony morphology varied depending on the substrate. On insoluble polysaccharides, the colonies were mostly pale-pink, thin, spreading, and irregular, up to 4 mm; on soluble sugars, the colonies were pink, more regular, and convex, up to 3 mm. The core membrane diether lipids are dominated by C_20_-C_20_ DGE (archaeol) and C_20_-C_25_ DGE (extended archaeol). The polar lipid head groups include phosphatidylglycerolphosphate methyl ester (PGP-Me) and phosphatidylglycerol (PG). The dominant respiratory menaquinone is MK-8:8, with the MK-8:7 second in abundance. The species are obligatory aerobic saccharolytic heterotrophs that are able to grow with insoluble beta-1,4-mannan, glucomannan, and cellulose. Sugars supporting the growth include galactose, rhamnose, raffinose, lactose, sucrose, cellobiose, maltose, trehalose, melezitose, and melibiose. Weak growth was observed on glycerol and pyruvate. Organic compounds tested (in the type strain) but not utilized included fructose, arabinose, ribose, xylose, sugar alcohols, acetate, ethanol, lactate, succinate, fumarate, malate, glycine, aspartate, glutamate, arginine, yeast extract, and peptone from casein. Ammonium and urea serve as the nitrogen source. Oxidase and catalase are positive. Mesophilic, with a maximum growth temperature of 48°C, is a low Mg-demanding, extreme halophile, with a range of Na^+^ for growth from 2.5 to 4.5 M (optimum at 3.5 M), and a facultative alkaliphile, with a pH range from 6.8 to 9.6 for growth (optimum at 9–9.2). The G + C content of the DNA is 62% (two genomes), and the habitat is hypersaline salt lakes. The type strain (AArc-m2/3/4^T^= JCM 34861 = UQM 41565) was isolated from sediments of hypersaline soda lakes in the Kulunda Steppe (Altai, Russia). The species also includes other four closely related isolates, one of which is deposited in JCM (AArc-xg1-1 = JCM 34866). The draft genome assemblies' accession numbers of strains AArc-m2/3/4^T^ and AArc-xg1-1 in the GenBank are GCA_025517485 and GCA_025517495.

## Data availability statement

The datasets presented in this study can be found in online repositories. The names of the repository/repositories and accession number(s) can be found in the article/Supplementary material.

## Author contributions

DS: Conceptualization, Investigation, Writing—original draft. AE: Investigation, Writing—original draft. NB: Investigation, Writing—original draft. JS: Writing—original draft. IK: Writing—original draft.
