# Supplementary material for: Natronoglomus mannanivorans gen. nov., sp. nov., beta-1,4-mannan utilizing natronoarchaea from hypersaline soda lakes
Source: Front Microbiol. 2024 Mar 12;15:1364606. doi: 10.3389/fmicb.2024.1364606 (PMC10963439; doi:10.3389/fmicb.2024.1364606)
Supplement: Supplementary file 1 [file Data_Sheet_1.PDF]

Natronoglomus mannanivorans gen. nov., sp. nov., beta-1,4-mannan utilizing natronoarchaea from hypersaline soda lakes

Dimitry Y. Sorokin[a,b*], Alexander G. Elcheninov[a], Nicole J. Bale[c], Jaap Sininghe-Damste[c] and Ilya V. Kublanov[a]

[a]*Winogradsky Institute of Microbiology, Research Centre of Biotechnology, Russian Academy of Sciences, Moscow, Russia*

[b]*Department of Biotechnology, Delft University of Technology, Delft, The Netherlands*

[c] *NIOZ Royal Netherlands Institute for Sea Research, Den Burg, Texel, The Netherlands*

Supplementary files

Table S1. Membrane polar lipids detected in strain AArc-m2/3/4.

Table S2. Whole genome comparison indexes between strains AArc-m2/3/4, AArc-xg1-1 and its closest representatives.

Table S3. Enzymes potentially involved in hydrolysis of various poly- and oligo-saccharides encoded in the genome of strain AArc-m2/3/4 (except for mannanases, cellulases and xylanases presented in the main text in **Table 1**).

Table S4. Complete repertoir of the CAZy enzymes encoded in the genomes of 2 mannan-utilizing natronoarchaea and closely related undescribed strains KZCA124 and TS33.

Table S5. A selection of functionally important proteins encoded in the genome of strain AArc-m2/3/4^T.

Fig. S1. Colonial growth of beta-mannan utilizing natronoarchaea on insoluble beta-1,4-mannan (left column) and amorphous cellulose (right column) showing hydrolysis clearance zones around the colonies**.** **a-b**, strain AArc-m2/3/4; **c-d**, strain AArc-gm4; **e-f**, strain Aarc-glctm5; **g-h**, strain HArc-m1. The medium contained 4 M total Na^+ at pH 9.5, incubation time – 10-14 d at 37°C.

Fig. S2. Maximum likelihood phylogenetic tree based on 122 conserved archaeal proteins demonstrating position of two strains of mannan-utilizing natronoarchaea (in bold) within the family *Natrialbaceae*. The branch lengths correspond to the number of substitutions per site with corrections associated with the models. The numbers at nodes indicate that the percentage of corresponding support values. *Archaeoglobus fulgidus* VC-16^T, *Methanocella paludicola* SANAE^T and *Methanothermobacter thermautotrophicus* Delta H^T were used as an outgroup (not shown).

Fig. S3. Phylogenetic analysis of enzymes of belonging to GH5 family. The numbers at nodes indicate that the percentage of corresponding support values (only values higher than 50% are shown). GH5_7 and H5_8 subfamilies are bounded with blue lines.

Table S1. Composition of intact polar lipids identified in strain AArc-m2/3/4^{T}

Polar head group	Core	$[M+H]^+$	Assigned elemental composition	% from total
**PGP-Me**	EXT-AR	971.7449	$C_{52}H_{109}O_{11}P_2$	10.1
	Uns(1)-EXT-AR	969.7286	$C_{52}H_{107}O_{11}P_2$	2.6
	Uns(2)-EXT-AR	967.7138	$C_{52}H_{105}O_{11}P_2$	1.3
	Uns(3)-EXT-AR	965.6976	$C_{52}H_{103}O_{11}P_2$	1.9
	Lyso-EXT-AR	691.3527	$C_{32}H_{69}O_{11}P_2$	0.6
	AR	901.6658	$C_{47}H_{99}O_{11}P_2$	30.7
	Lyso-AR	621.3527	$C_{27}H_{59}O_{11}P_2$	1.8
	**Total**			**49.0**
**PG**	EXT-AR	877.7623	$C_{51}H_{106}O_8P$	14.3
	Uns(1)-EXT-AR	875.7461	$C_{51}H_{104}O_8P$	2.5
	AR	807.6837	$C_{46}H_{96}O_8P$	31.7
	Lyso-AR	527.3704	$C_{26}H_{56}O_8P$	2.1
	**Total**			**50.6**
**PG-Gly**	Ext-AR	1039.815	$C_{57}H_{116}O_{13}P$	**0.4**
	**Sum AR**			**27**
	**Sum EXT-AR**			**32**
	**Sum uns-EXT-AR**			**38**
	**Sum lyso-AR**			**3.3**

PGP-Me = phosphatidylglycerolphosphate methyl ester; PG = phosphatidylglycerol; PG-Gly= phosphatidylglycerohexose; AR = archaeol; (C_{20}-C_{20}); EXT-AR = extended archaeol (C_{20}-C_{25}); lyso = one alkyl chain is absent; uns = unsaturated.

Supplementary Table 2. ANI values between strains AArc-m2/3/4, AArc-xg1-1 and type species of the *Natrialbaceae* family

Species	GCF_025629245	GCA_000025325	GCA_000217715	GCA_000230715	GCA_000230735	GCA_000328685	GCA_000337215	GCA_000337515	GCA_000337555	GCA_000517625	GCA_002177135	GCA_003430825	GCA_003841505	GCA_004799645	GCA_006543045	GCA_017357405	GCA_020177375	GCA_020567525	GCA_023008585	GCA_023566055	GCA_024138145	GCA_024296665	GCA_024362485	GCA_024362525	**GCA_025517485**	**GCA_025517495**	GCA_029338335	GCA_900103505	GCA_900112205
GCF_025629245.1_Natronobiforma_cellulositrpha_AArcel5	100	77.21	77.37	76.78	77.16	76.98	77.2	75.93	76.66	76.59	76.16	76.79	77.24	76.27	76.57	78.3	77.3	74.44	78.27	77.29	77.06	78.08	78.3	77.9	**78.07**	**78.07**	78.2	75.36	77.2
GCA_000025325.1_Haloterrigena_turkmenica_DSM_5511	77.07	100	80.7	78.51	80.67	79.14	80.69	76.37	78.89	77.86	78	78.47	78.6	79.22	78.64	78.16	77.11	74.53	78.54	79.97	77.38	78.84	77.88	78.1	**77.79**	**77.8**	78.16	75.71	79.52
GCA_000217715.1_Halopiger_xanaduensis_SH-6	77.21	80.7	100	79.08	80.29	79.45	80.07	76.63	79.04	77.79	78.7	78.54	78.93	78.55	78.84	78.26	77.56	74.93	78.9	79.95	77.63	79.12	78.49	78.58	**77.99**	**78.03**	78.22	76.12	80.37
GCA_000230715.3_Natronobacterium_gregoryi_SP2	76.67	78.72	79.01	100	78.69	78.49	78.83	75.71	77.95	76.99	77.89	78.15	78.74	77.89	78.13	77.37	76.51	74.19	78.2	78.9	76.79	77.7	77.72	77.5	**77.3**	**77.32**	77.24	74.96	80.58
GCA_000230735.3_Natrinema_pellirubrum_DSM_15624	76.92	80.79	80.11	78.66	100	78.99	79.47	76.43	78.67	77.77	77.81	78.08	78.57	78.62	79.01	78	77.55	74.46	79.27	79.81	77.35	78.8	77.95	78.07	**77.45**	**77.52**	77.7	75.7	79.79
GCA_000328685.1_Natronococcus_occultus_SP4	76.73	79.26	79.37	78.41	79.1	100	79.08	76.18	78.02	77.25	77.19	78.02	78.42	77.83	78.29	77.87	76.96	74.63	78.35	79.29	77.21	78.5	77.98	78.36	**77.45**	**77.46**	77.76	75.61	79.05
GCA_000337215.1_Natronolimnohabitans_innermongolicus_JCM_12255	77.43	80.81	79.94	78.79	79.74	79.14	100	76.44	78.71	77.89	78.37	79.01	78.8	79.33	79.27	78.08	76.95	74.56	78.84	79.64	77.49	78.81	78.35	78.44	**77.79**	**77.82**	78.11	75.82	79.35
GCA_000337515.1_Halovivax_asiaticus_JCM_14624	75.78	76.39	76.53	75.59	76.35	75.98	76.37	100	75.96	75.79	75.12	75.77	76.05	75.34	75.79	76.17	75.58	74.15	76.28	76.51	75.98	76.72	76.04	76.11	**75.74**	**75.74**	76.11	74.92	76.35
GCA_000337555.1_Natrialba_asiatica_DSM_12278	76.53	79.1	78.94	77.89	78.78	78.14	78.71	75.84	100	77.13	77.52	77.59	78.12	77.89	78.1	77.47	76.11	74.16	77.86	78.78	76.86	77.73	77.15	77.42	**77.02**	**77.05**	77.4	75.16	78.56
GCA_000517625.1_Halostagnicola_larsenii_XH-48	76.47	78.04	77.65	76.92	77.81	77.24	77.87	75.63	77.36	100	76.6	77.02	77.54	77.06	77.2	77.22	75.96	73.88	77.62	77.77	76.92	77.39	77.38	77.3	**76.99**	**77.02**	77.2	74.8	77.57
GCA_002177135.1_Natronolimnobius_baerhuensis_CGMCC_1.3597	75.88	78.05	78.64	77.74	78.07	77.29	78.39	75.24	77.66	76.7	100	77.2	77.64	77.61	77.9	76.65	75.6	73.78	77.14	77.83	76.15	76.72	76.74	76.59	**76.52**	**76.52**	76.63	74.56	77.95
GCA_003430825.1_Natrarchaeobaculum_sulfurireducens_AArc1	77.01	78.73	78.6	78.25	78.46	78.32	79.12	75.9	77.71	77.11	77.62	100	78.48	77.95	78.35	77.29	76.56	74.16	78.36	78.67	77.05	77.89	78.02	77.77	**77.27**	**77.34**	77.28	74.97	78.5
GCA_003841505.1_Natrarchaeobius_chitinivorans_AArcht4	76.89	78.56	78.76	78.55	78.53	78.41	78.76	76.16	78.27	77.37	77.56	78.33	100	78.11	78.28	77.44	76.65	74.22	78.47	78.98	76.89	78.16	77.95	77.77	**77.16**	**77.27**	77.22	74.95	78.81
GCA_004799645.1_Natronorubrum_bangense_JCM_10635	76.39	79.39	78.58	78.09	78.62	78.05	79.37	75.75	77.92	77.12	77.78	78.05	78.08	100	78.08	77.23	76	73.88	77.89	78.9	76.67	77.42	78.02	77.2	**77.13**	**77.3**	77.23	74.72	78.17
GCA_006543045.1_Salinadaptatus_halalkaliphilus_XQ-INN	76.37	78.62	78.75	77.98	78.78	78.2	79.17	75.7	78.14	77.14	77.89	78.24	78.48	77.96	100	77.1	76.47	73.61	77.77	78.48	76.48	77.41	77.1	77.22	**76.97**	**76.93**	76.99	74.5	78.55
GCA_017357405.1_KZCA124	78.03	77.99	78.06	77.47	78.07	77.64	78.2	76.11	77.21	77.09	76.51	77.19	77.44	77.22	77.27	100	77.43	74.32	78.87	77.97	77.33	78.53	78.09	78.19	**85.59**	**85.72**	96.87	75.33	77.91
GCA_020177375.1_Saliphagus_infecundisoli_YIM_93745	76.8	76.7	76.99	76.13	77.07	76.78	76.8	75.35	75.79	75.69	75.27	76.16	76.27	75.56	76.12	77.31	100	73.84	77.05	76.88	75.83	77.3	76.91	76.92	**76.92**	**76.89**	77.3	74.94	77.16
GCA_020567525.1_Halorubellus_salinus_GX3	74.29	74.73	74.85	73.88	74.53	74.55	74.77	74.11	74.2	73.97	73.87	74.04	74.11	73.72	73.8	74.39	74.07	100	74.7	74.55	74.29	74.99	74.48	74.57	**74.36**	**74.4**	74.38	76.5	74.64
GCA_023008585.1_Natribaculum_breve_TRM20010	77.99	78.66	78.82	78.11	79.05	78.28	78.76	76.33	77.88	77.56	77	77.97	78.19	77.55	78.03	78.63	77.39	74.7	100	79.32	77.77	79.98	80.5	79.04	**78.41**	**78.52**	78.68	75.67	78.74
GCA_023566055.1_Halosolutus_amylolyticus_WLHS5	77.01	79.94	79.97	78.8	79.87	79.29	79.68	76.37	78.79	77.75	77.7	78.47	78.93	78.8	78.41	77.98	77.02	74.66	79.12	100	77.48	79.19	78.43	78.21	**77.68**	**77.74**	77.95	75.5	79.56
GCA_024138145.1_Natronosalvus_halobius_AGai3-5	77.05	77.41	77.59	76.66	77.37	77.23	77.59	75.94	76.93	76.75	76.16	76.86	76.97	76.62	76.62	77.45	76.25	74.35	77.96	77.65	100	78.08	77.75	77.8	**77.21**	**77.29**	77.44	75.29	77.41
GCA_024296665.1_Salinilacihabitans_rarus_AD-4	78	79.02	79.16	77.88	78.82	78.67	78.96	76.74	77.9	77.45	76.83	77.84	78.15	77.2	77.68	78.66	77.59	75.12	80	79.39	78.15	100	79.38	79.42	**78.25**	**78.31**	78.69	76.42	79.21
GCA_024362485.1_Natronobeatus_ordinarius_WLHSJ27	77.94	77.94	78.24	77.46	77.76	77.85	78.12	75.98	77.1	77.08	76.61	77.8	77.71	77.74	77.12	78	77.03	74.54	80.31	78.38	77.61	79.13	100	78.65	**77.85**	**77.9**	77.94	75.45	77.94
GCA_024362525.1_Natrononativus_amylolyticus_CGA73	77.93	78.22	78.29	77.36	78.12	78.27	78.39	76.15	77.54	77.28	76.65	77.55	77.8	77.2	77.4	78.28	77.23	74.63	78.87	78.24	77.85	79.26	78.68	100	**77.89**	**77.95**	78.19	75.66	78.11
**GCA_025517485.1_Natronoglomus_mannanivorans_AArc-m234**	**77.88**	**77.59**	**77.73**	**77.05**	**77.48**	**77.34**	**77.7**	**75.8**	**76.89**	**76.8**	**76.44**	**76.83**	**77.16**	**76.81**	**76.95**	**85.48**	**77.12**	**74.38**	**78.58**	**77.76**	**77.07**	**77.97**	**78**	**77.92**	**100**	**98.56**	**85.64**	**75.12**	**77.62**
**GCA_025517495.1_Natronoglomus_mannanivorans_AArc-xg1-1**	**77.9**	**77.64**	**77.69**	**77.01**	**77.52**	**77.33**	**77.75**	**75.82**	**76.84**	**76.96**	**76.38**	**76.94**	**77.18**	**77.14**	**76.99**	**85.61**	**77.03**	**74.13**	**78.49**	**77.62**	**77.03**	**78.05**	**78**	**77.83**	**98.53**	**100**	**85.7**	**75.18**	**77.56**
GCA_029338335.1_TS33	77.76	77.91	77.8	77.09	77.82	77.43	77.88	75.88	77.19	76.98	76.49	77.08	77.09	76.91	77.1	96.4	77.36	74.33	78.72	77.76	77.09	78.41	77.94	78	**85.4**	**85.49**	100	75.45	77.78
GCA_900103505.1_Haloarchaeobius_iranensis_EB21	75.38	75.91	76.07	74.95	75.8	75.5	75.84	74.88	75.29	74.83	74.52	74.95	75.24	74.65	75	75.39	75.11	76.38	76.01	75.78	75.42	76.26	75.63	75.78	**75.32**	**75.46**	75.59	100	75.92
GCA_900112205.1_Halobiforma_haloterrestris_DSM_13078	76.95	79.46	80.19	80.37	79.53	79.21	79.46	76.23	78.45	77.27	77.92	78.28	78.56	78.07	78.55	77.82	77.2	74.64	78.7	79.64	77.41	78.89	77.98	78.11	**77.53**	**77.4**	77.69	75.68	100

Supplementary Table 2b. AAI values between strains AArc-m2/3/4, AArc-xg1-1 and type species of the *Natrialbaceae* family

Species	GCF_025629245	**GCA_025517485.1**	**GCA_025517495.1**	GCF_000025325.1	GCF_000217715.1	GCF_000230715.2	GCF_000230735.2	GCF_000328685.1	GCF_000337215.1	GCF_000337515.1	GCF_000337555.1	GCF_000517625.1	GCF_002177135.1	GCF_003430825.1	GCF_003841505.1	GCF_004799645.1	GCF_006543045.1	GCF_017357405.1	GCF_020177375.1	GCF_020567525.1	GCF_023008585.1	GCF_023566055.1	GCF_024138145.1	GCF_024296665.1	GCF_024362485.1	GCF_024362525.1	GCF_029338335.1	GCF_900103505.1	GCF_900112205
GCF_025629245.1_Natronobiforma_cellulositropha_AArcel5	100	**70.33**	**70.34**	66.26	66.71	66.7	66.61	66.76	66.72	63.57	65.49	66.77	66.51	67.29	67.26	67.25	65.99	69.74	67.05	59.06	68.39	66.84	66.7	68.67	68.83	68.61	70.01	60.91	66.35
**GCA_025517485.1_Natronoglomus_mannanivorans_AArc-m234**	70.33	**100**	**98.32**	**67.53**	**68.25**	**67.59**	**67.75**	**67.62**	**67.5**	**64.09**	**66.92**	**67.99**	**67.4**	**68.07**	**67.9**	**68.16**	**66.78**	**85.05**	**68.03**	**59.09**	**69.97**	**68.26**	**67.35**	**69.48**	**69.08**	**69.49**	**84.72**	**60.86**	**67.45**
**GCA_025517495.1_Natronoglomus_mannanivorans_AArc-xg1-1**	70.34	**98.32**	**100**	**67.59**	**68.23**	**67.52**	**67.96**	**67.79**	**67.75**	**64.11**	**67.12**	**67.78**	**67.44**	**67.87**	**67.74**	**68.18**	**66.82**	**85.28**	**68.18**	**58.9**	**70.18**	**68.3**	**67.69**	**69.68**	**69.16**	**69.53**	**84.79**	**60.89**	**67.52**
GCF_000025325.1_Haloterrigena_turkmenica_DSM_5511	66.26	**67.53**	**67.59**	100	74.1	71.44	75.09	71.5	75.59	64.75	71.55	70.21	70.77	71.4	70.38	74.58	71.04	67.88	65.73	59.73	69.99	73.11	67.24	69.7	68.37	68.35	67.86	61.61	71.92
GCF_000217715.1_Halopiger_xanaduensis_SH-6	66.71	**68.25**	**68.23**	74.1	100	72.25	73.38	71.53	72.02	64.87	71.75	69.93	74.01	71.92	71.83	72.15	71.17	68.58	66.56	59.69	70.16	72.68	67.04	69.96	68.84	68.48	68.41	61.4	73.67
GCF_000230715.2_Natronobacterium_gregoryi_SP2	66.7	**67.59**	**67.52**	71.44	72.25	100	70.95	71.71	71.74	64.21	70.7	68.4	71.71	72.48	72	72.29	71.67	67.32	65.92	59.96	69.46	71.69	66.89	69.25	69.01	68.96	67.17	61.49	76.69
GCF_000230735.2_Natrinema_pellirubrum_DSM_15624	66.61	**67.75**	**67.96**	75.09	73.38	70.95	100	71.04	72.62	64.94	71.55	70.04	70.72	71.58	70.73	72.95	71.19	68.42	66.68	60.24	70.46	73.41	67.61	70.16	68.36	68.78	68.2	62.23	72.23
GCF_000328685.1_Natronococcus_occultus_SP4	66.76	**67.62**	**67.79**	71.5	71.53	71.71	71.04	100	71.77	64.46	70.45	69.11	70.73	71.61	70.4	71.76	70.51	67.56	66.07	60.29	69.2	71.57	67.26	69.43	68.65	69.28	67.25	61.53	71.45
GCF_000337215.1_Natronolimnohabitans_innermongolicus_JCM_12255	66.72	**67.5**	**67.75**	75.59	72.02	71.74	72.62	71.77	100	63.76	70.27	69.05	71.78	72.36	71.09	74.95	72.2	67.03	65.6	59.12	69.14	71.86	66.52	68.93	68.56	68.46	66.86	60.97	71.14
GCF_000337515.1_Halovivax_asiaticus_JCM_14624	63.57	**64.09**	**64.11**	64.75	64.87	64.21	64.94	64.46	63.76	100	64.43	64.15	63.63	64.55	63.96	64.12	63.63	64.41	63.83	59.39	65.75	65.34	64.58	66	65.15	65.18	64.2	60.44	64.68
GCF_000337555.1_Natrialba_asiatica_DSM_12278	65.49	**66.92**	**67.12**	71.55	71.75	70.7	71.55	70.45	70.27	64.43	100	69.01	69.97	70.52	70.7	70.82	70.31	67.07	65.36	59.09	68.66	70.93	66.2	68.76	67.59	67.82	66.84	60.9	71.54
GCF_000517625.1_Halostagnicola_larsenii_XH-48	66.77	**67.99**	**67.78**	70.21	69.93	68.4	70.04	69.11	69.05	64.15	69.01	100	68.37	68.73	68.32	69.48	68.27	67.68	65.91	59.7	68.66	68.97	67.03	69.03	68.18	68.02	67.7	61.11	69.04
GCF_002177135.1_Natronolimnobius_baerhuensis_CGMCC_1.3597	66.51	**67.4**	**67.44**	70.77	74.01	71.71	70.72	70.73	71.78	63.63	69.97	68.37	100	71.48	71	71.6	71.23	67.28	65.6	58.9	68.17	70.35	66.18	67.99	67.94	67.86	67.07	60.34	71.48
GCF_003430825.1_Natrarchaeobaculum_sulfurireducens_AArc1	67.29	**68.07**	**67.87**	71.4	71.92	72.48	71.58	71.61	72.36	64.55	70.52	68.73	71.48	100	72.55	72.46	73.58	67.59	66.15	59.69	69.58	71.17	67.2	69.57	69.94	69.44	67.45	61.15	71.68
GCF_003841505.1_Natrarchaeobius_chitinivorans_AArcht4	67.26	**67.9**	**67.74**	70.38	71.83	72	70.73	70.4	71.09	63.96	70.7	68.32	71	72.55	100	71.68	71.55	67.07	65.7	58.9	68.97	71.16	66.78	69.21	68.68	68.81	67.1	60.98	71.32
GCF_004799645.1_Natronorubrum_bangense_JCM_10635	67.25	**68.16**	**68.18**	74.58	72.15	72.29	72.95	71.76	74.95	64.12	70.82	69.48	71.6	72.46	71.68	100	71.69	67.94	65.54	59.45	69.5	72.97	67.37	69.55	69.68	69.26	67.89	61.32	71.74
GCF_006543045.1_Salinadaptatus_halalkaliphilus_XQ-INN	65.99	**66.78**	**66.82**	71.04	71.17	71.67	71.19	70.51	72.2	63.63	70.31	68.27	71.23	73.58	71.55	71.69	100	66.87	65.3	58.86	68.11	70.44	66.13	67.98	67.6	67.78	66.43	60.62	71.26
GCF_017357405.1_KZCA124_protein	69.74	**85.05**	**85.28**	67.88	68.58	67.32	68.42	67.56	67.03	64.41	67.07	67.68	67.28	67.59	67.07	67.94	66.87	100	68.68	59.6	70.18	68.01	67.93	69.98	68.57	69.48	96.44	61.43	67.59
GCF_020177375.1_Saliphagus_infecundisoli_YIM_93745	67.05	**68.03**	**68.18**	65.73	66.56	65.92	66.68	66.07	65.6	63.83	65.36	65.91	65.6	66.15	65.7	65.54	65.3	68.68	100	59.62	67.17	65.82	66.12	67.67	66.94	67.04	68.63	61.24	66.55
GCF_020567525.1_Halorubellus_salinus_GX3	59.06	**59.09**	**58.9**	59.73	59.69	59.96	60.24	60.29	59.12	59.39	59.09	59.7	58.9	59.69	58.9	59.45	58.86	59.6	59.62	100	61.28	60.24	59.65	61.57	60.06	60.44	59.18	64.45	60
GCF_023008585.1_Natribaculum_breve_TRM20010	68.39	**69.97**	**70.18**	69.99	70.16	69.46	70.46	69.2	69.14	65.75	68.66	68.66	68.17	69.58	68.97	69.5	68.11	70.18	67.17	61.28	100	70.93	69.1	72.66	73.38	71.02	70.06	62.75	70.22
GCF_023566055.1_Halosolutus_amylolyticus_WLHS5	66.84	**68.26**	**68.3**	73.11	72.68	71.69	73.41	71.57	71.86	65.34	70.93	68.97	70.35	71.17	71.16	72.97	70.44	68.01	65.82	60.24	70.93	100	67.96	71.19	69.17	68.95	67.96	61.73	72.11
GCF_024138145.1_Natronosalvus_halobius_AGai3-5	66.7	**67.35**	**67.69**	67.24	67.04	66.89	67.61	67.26	66.52	64.58	66.2	67.03	66.18	67.2	66.78	67.37	66.13	67.93	66.12	59.65	69.1	67.96	100	69.68	68.22	69.23	67.31	61.63	67.43
GCF_024296665.1_Salinilacihabitans_rarus_AD-4	68.67	**69.48**	**69.68**	69.7	69.96	69.25	70.16	69.43	68.93	66	68.76	69.03	67.99	69.57	69.21	69.55	67.98	69.98	67.67	61.57	72.66	71.19	69.68	100	71.3	71.71	69.81	63.09	70.59
GCF_024362485.1_Natronobeatus_ordinarius_WLHSJ27	68.83	**69.08**	**69.16**	68.37	68.84	69.01	68.36	68.65	68.56	65.15	67.59	68.18	67.94	69.94	68.68	69.68	67.6	68.57	66.94	60.06	73.38	69.17	68.22	71.3	100	70.74	68.31	61.82	68.85
GCF_024362525.1_Natrononativus_amylolyticus_CGA73	68.61	**69.49**	**69.53**	68.35	68.48	68.96	68.78	69.28	68.46	65.18	67.82	68.02	67.86	69.44	68.81	69.26	67.78	69.48	67.04	60.44	71.02	68.95	69.23	71.71	70.74	100	69.28	62.1	68.86
GCF_029338335.1_TS33_protein	70.01	**84.72**	**84.79**	67.86	68.41	67.17	68.2	67.25	66.86	64.2	66.84	67.7	67.07	67.45	67.1	67.89	66.43	96.44	68.63	59.18	70.06	67.96	67.31	69.81	68.31	69.28	100	60.99	67.44
GCF_900103505.1_Haloarchaeobius_iranensis_EB21	60.91	**60.86**	**60.89**	61.61	61.4	61.49	62.23	61.53	60.97	60.44	60.9	61.11	60.34	61.15	60.98	61.32	60.62	61.43	61.24	64.45	62.75	61.73	61.63	63.09	61.82	62.1	60.99	100	61.36
GCF_900112205.1_Halobiforma_haloterrestris_DSM_13078	66.35	**67.45**	**67.52**	71.92	73.67	76.69	72.23	71.45	71.14	64.68	71.54	69.04	71.48	71.68	71.32	71.74	71.26	67.59	66.55	60	70.22	72.11	67.43	70.59	68.85	68.86	67.44	61.36	100

Supplementary Table 2c. POCP values between strains AArc-m2/3/4, AArc-xg1-1 and type species of *the Natrialbaceae* family

Species	GCF_000337555.1	GCF_025629245_N	**GCA_025517485.1**	**GCA_025517495.1**	GCF_000025325.1	GCF_000217715.1	GCF_000230715.2	GCF_000230735.2	GCF_000328685.1	GCF_000337215.1	GCF_000337515.1	GCF_000517625.1	GCF_002177135.1	GCF_003430825.1	GCF_003841505.1	GCF_004799645.1_Natronorubrum_bangense_JCM_10635													
GCF_000337555.1_Natrialba_asiatica_DSM_12278	100.00	56.92	**60.88**	**60.73**	70.01	70.38	63.13	65.87	65.20	65.16	59.55	70.56	66.02	63.35	64.06	64.11	GCF_006543045.1	GCF_017357405.1	GCF_020177375.1	GCF_020567525.1	GCF_023008585.1	GCF_023566055.1	GCF_024138145.1	GCF_024296665.1	GCF_024362485.1	GCF_024362525.1	GCF_029338335.1	GCF_900103505.1	GCF_900112205
GCF_025629245.1_Natronobiforma_cellulositropha_AArcel5		100.00	**66.33**	**66.03**	58.57	60.09	58.72	59.98	60.11	60.50	56.06	58.95	64.14	59.40	62.53	61.37	63.32	61.87	59.15	50.26	63.12	67.25	64.32	63.32	62.07	63.59	61.38	53.37	67.81
**GCA_025517485.1_Natronoglomus_mannanivorans_AArc-m234**			**100.00**	**92.47**	**63.53**	**65.04**	**56.41**	**61.37**	**61.81**	**62.17**	**54.87**	**61.68**	**66.65**	**58.10**	**63.78**	**60.95**	61.68	63.89	58.60	48.95	60.41	60.18	61.36	60.82	61.77	62.54	63.11	51.49	58.86
**GCA_025517495.1_Natronoglomus_mannanivorans_AArc-xg1-1**				**100.00**	**63.37**	**64.63**	**56.59**	**61.87**	**61.25**	**62.05**	**54.67**	**60.84**	**66.22**	**58.09**	**64.10**	**60.89**	**60.52**	**80.35**	**63.47**	**49.67**	**60.26**	**63.73**	**63.59**	**61.71**	**61.85**	**65.20**	**80.49**	**51.49**	**61.75**
GCF_000025325.1_Haloterrigena_turkmenica_DSM_5511					100.00	73.44	60.93	71.09	69.67	71.28	57.65	69.13	65.19	62.76	64.70	68.34	**60.52**	**79.76**	**63.33**	**49.42**	**60.40**	**63.59**	**63.36**	**61.23**	**61.60**	**64.87**	**80.01**	**51.30**	**61.38**
GCF_000217715.1_Halopiger_xanaduensis_SH-6						100.00	64.58	69.88	66.88	66.89	59.75	68.41	72.81	66.05	66.40	66.82	64.43	65.71	60.43	51.87	64.72	72.57	64.62	64.76	63.60	63.62	65.20	53.70	68.41
GCF_000230715.2_Natronobacterium_gregoryi_SP2							100.00	64.45	66.78	66.71	59.56	61.72	67.38	75.11	67.75	69.24	65.65	66.11	60.91	52.43	64.78	70.77	64.78	66.12	63.84	62.90	66.35	54.75	71.24
GCF_000230735.2_Natrinema_pellirubrum_DSM_15624								100.00	65.57	69.68	60.89	65.42	65.40	66.23	66.12	70.27	67.93	55.60	54.13	50.60	64.72	66.74	60.84	65.36	67.17	62.05	54.05	53.65	71.61
GCF_000328685.1_Natronococcus_occultus_SP4									100.00	71.14	57.92	64.68	67.24	67.39	66.77	70.45	67.73	60.93	59.24	53.29	68.84	70.90	64.36	65.64	66.50	62.63	60.45	57.81	69.14
GCF_000337215.1_Natronolimnohabitans_innermongolicus_JCM_12255										100.00	58.28	65.51	68.63	70.74	68.39	74.33	65.74	60.31	59.90	50.73	63.90	69.76	63.91	64.79	67.44	68.16	59.08	52.40	68.92
GCF_000337515.1_Halovivax_asiaticus_JCM_14624											100.00	59.19	58.51	58.72	58.24	57.93	70.69	59.68	55.87	50.06	64.92	70.69	63.22	63.31	67.08	64.50	59.14	52.55	68.17
GCF_000517625.1_Halostagnicola_larsenii_XH-48												100.00	66.25	63.05	63.59	64.07	58.28	55.30	53.31	52.10	60.32	61.90	62.07	61.25	60.40	59.77	54.08	55.20	61.53
GCF_002177135.1_Natronolimnobius_baerhuensis_CGMCC_1.3597													100.00	68.41	69.68	68.33	63.20	62.48	60.66	50.70	62.88	64.96	63.20	62.61	62.62	63.26	61.55	52.70	65.00
GCF_003430825.1_Natrarchaeobaculum_sulfurireducens_AArc1														100.00	69.07	73.22	67.92	64.27	59.60	49.60	61.34	66.17	63.89	62.07	64.48	64.07	63.59	51.62	67.54
GCF_003841505.1_Natrarchaeobius_chitinivorans_AArcht4															100.00	67.74	71.52	56.20	53.61	51.23	65.04	67.58	61.95	64.06	69.39	63.62	55.27	54.31	70.58
GCF_004799645.1_Natronorubrum_bangense_JCM_10635																100.00	69.76	60.20	57.70	50.27	63.41	67.94	64.00	62.97	68.19	65.20	60.77	53.49	68.95
GCF_006543045.1_Salinadaptatus_halalkaliphilus_XQ-INN																	100.00	58.24	55.80	50.04	63.04	66.80	60.62	61.43	66.46	62.02	57.36	52.32	69.41
GCF_017357405.1_KZCA124_protein																		100.00	64.81	50.64	60.17	63.40	64.72	62.42	59.65	64.84	88.23	52.77	61.62
GCF_020177375.1_Saliphagus_infecundisoli_YIM_93745																			100.00	50.02	59.85	59.29	61.49	59.34	57.85	62.83	64.34	52.24	57.90
GCF_020567525.1_Halorubellus_salinus_GX3																				100.00	55.49	56.28	55.33	56.74	52.95	53.43	49.29	65.71	54.54
GCF_023008585.1_Natribaculum_breve_TRM20010																					100.00	69.85	64.84	68.83	71.40	64.60	60.05	58.87	67.07
GCF_023566055.1_Halosolutus_amylolyticus_WLHS5																						100.00	67.76	71.30	68.24	66.04	63.49	58.03	74.54
GCF_024138145.1_Natronosalvus_halobius_AGai3-5																							99.97	67.88	65.08	70.38	63.69	57.15	65.39
GCF_024296665.1_Salinilacihabitans_rarus_AD-4																								100.00	66.80	67.19	60.47	58.43	69.89
GCF_024362485.1_Natronobeatus_ordinarius_WLHSJ27																									99.98	67.40	59.13	56.54	68.73
GCF_024362525.1_Natrononativus_amylolyticus_CGA73																										100.00	63.68	54.78	65.40
GCF_029338335.1_TS33_protein																											100.00	51.36	60.97
GCF_900103505.1_Haloarchaeobius_iranensis_EB21																												100.00	57.22
GCF_900112205.1_Halobiforma_haloterrestris_DSM_13078																													100.00

Table S3.

Locus tag MCU4972+	Protein family	Putative function	Localization (signal peptide)
1417	GH3	beta-glycosidase	C
1587	GH105	alpha-rhamnogalacturonyl hydrolase	C
1683<br>1684	GH15<br>GH13_20	Glucoamylase<br>Maltogenic alpha-amylase	C<br>C
2278<br>2279	GH5<br>GH31_4	Putative xyloglucan-specific glucanase<br>alpha-xylosidase	E (TAT/SPI)<br>C
2298	GH67	alpha-glucuronidase	C
2305	GH3	beta-glycosidase	C
2307<br>2308	GH4<br>GH10	alpha-glycosidase<br>beta-1,4-xylanase	C<br>C
2327<br>2328	GH42<br>PL42	beta-galactosidase<br>rhamnose-alpha-1,4-glucuronate lyase	C<br>C
2490	GH43_3/CBM13	beta-galactofuranosidase	E (TAT/SPI)
2564	GH3	beta-glycosidase	C
3085	GH15	glucoamylase	C
3255	GH93	exo-alpha-arabinofuranosidase	C
3417<br>3418	GH51_1<br>GH2	arabinan-exo-alpha-1,3- arabinofuranosidase<br>alpha-arabinopyranosidase	C<br>C
3421	GH43_3	beta-galactofuranosidase	E (Sec-TAT/SPII)
3427	GH2	alpha- arabinopyranosidase	C
3431	GH127	beta-arabinopyranosidase	C
3461	GH28	alpha-galacturonase	C
3474<br>3476	PL1_2<br>PL1_2	Pectate lyase	E (TAT/SPI)
3506	CBM13	Xylan-binding domain	E (TAT/SPI)
3507<br>3508	GH2<br>GH2	beta-glycosidase	E (TAT/SPI)<br>C
3509	PL26	Rhamnogalacturonan exo-lyase	C
3513	GH28	alpha-galacturonase	C
3532<br>3533	PL22<br>2 x PL22	Oligogalacturonate lyase	C
3537	GH78/CBM67	Rhamnogalacturonan alpha-rhamnosidase	C
3538	CBM13	Carbohydrate-binding domain	E (TAT/SPI)
3540<br>3541	GH51_1<br>GH106	Arabinan-exo-alpha-1,3- arabinofuranosidase<br>Rhamnogalacturonan alpha-rhamnohydrolase	C<br>C
3543	2 x PL22	Oligogalacturonate lyase	C
3545	GH43	alpha-arabinanase	C
3549	GH95	alpha-galacto/fucosidase	C
3550	GH43_18	alpha-arabinofuranosidase	C
3552	PL11_1	Rhamnogalacturonan lyase	C
3555<br>3556; 3558	GH78/CBM67<br>GH2	Rhamnogalacturonan alpha-rhamnosidase<br>beta-galacturonidases	C<br>C
3562	GH42	beta-galactosidase	C
3563	2 x CBM13	Carbohydrate-binding domain	E (TAT/SPI)
3677	GH43_12/CBM91	beta-xylosidase	C
3844	GH32	Levansucrase/inulinase	C

3845	GH154	beta-1,6-glucuronidases	C
3874	CBM88	Xyloglucan/galactomannan-specific CBM	E (TAT/SPI)
4048; 4050; 4057	GH2	beta-galactosidases	C
4079	GH88	beta-glucuronyl hydrolase	C
4103<br>4104	GH95<br>GH29	alpha-fucosidase/galactosidase<br>alpha-fucosidase/galactosidase	C
4109	GH2	beta-galactosidase	C
4265	GH3	beta-glucosidase	C
4344<br>4345	GH43_12/CBM91<br>GH159	beta-xylosidase<br>alpha-arabinofuranosidase	C
4390	GH4	alpha-gluco/galactosidase	C
5260	GH51_1	arabinan-exo-alpha-1,3- arabinofuranosidase	E (TAT/SPII)
5300<br>5301<br>5302<br>5303<br>5305	GH28<br>2 x PL22<br>CBM9<br>GH115<br>GH4	alpha-galacturonase<br>Oligogalacturonate lyase<br>Cellulose-binding domain<br>alpha-1,6-glucuronidase<br>alpha-galacturonidase	C<br>C<br>C<br>C<br>C
5308<br>5309	GH78/CBM67<br>CBM13	Rhamnogalacturonan alpha-rhamnosidase<br>Carbohydrate-binding domain	C<br>E (TAT/SPI)
5450	GH88	Xyloglucan/galactomannan-specific CBM	C
5456<br>5457	GH29<br>GH137	alpha-fucosidase/galactosidase<br>beta-arabinofuranosidase	C<br>C
5461<br>5462	2 x GH2<br>GH2	beta-galactosidases	C
5469<br>5470	GH29<br>GH2	alpha-fucosidase/galactosidase<br>beta-galactosidases	C<br>C
5481<br>5482<br>5485; 5491	GH78/CBM67<br>GH106<br>GH78/CBM67	Rhamnogalacturonan alpha-rhamnosidase<br>Rhamnogalacturonan alpha-rhamnohydrolase<br>Rhamnogalacturonan alpha-rhamnosidase	C<br>C<br>C
5519	GH43_3/CBM13	endo-alpha-arabininase	E (TAT/SPI)
5556	GH3	beta-glycosidase	C
5789	GH95	alpha-fucosidase/galactosidase	C
5961;5962; 5964	GH29	alpha-fucosidase/galactosidase	C

(E), extracellular; (C), cytoplasmic

Table S4.

AArc-M2/3/4

Gene ID	HMMER	dbCAN_sub	DIAMOND	Function
MCU4971356.1	GH109(6-273)	GH109_e7	-	*N-* acetylgalactosaminidase
MCU4971437.1	GH3(52-260)	GH3_e103	GH3	beta-glucosidase
MCU4971528.1	GH109(5-276)	GH109_e2	-	*N-* acetylgalactosaminidase
MCU4971587.1	GH105(40-342)	GH105_e35	GH105	unsaturated rhamnogalacturonyl hydrolase
MCU4971683.1	GH15(283-639)	GH15_e46	GH15	putative GH15
MCU4971684.1	GH13_20(292-598)	GH13_e49	GH13	alpha-amylase
MCU4972278.1	GH5(67-371)	GH5_e197	GH5	endoglucanase
MCU4972279.1	GH31(221-716)	GH31_e10	GH31	alpha-xylosidase
MCU4972295.1	GH11(76-245)+CBM13(291-380)+CBM13(358-426)	GH11_e15+CBM13_e122	CBM13+GH11	endo-1,4-beta-xylanase
MCU4972296.1	CBM6(2-87)	CBM6_e65	-	xylan-binding CBM
MCU4972297.1	CBM85(106-238)+CBM85(329-460)+GH10(518-835)	CBM85_e8+CBM85_e8+GH10_e16	CBM85+GH10	endo-1,4-beta-xylanase
MCU4972298.1	GH67(8-688)	GH67_e1	GH67	alpha-1,2-glucuronosidase
MCU4972305.1	GH3(66-292)	GH3_e88	GH3	beta-xylosidase/beta-glucosidase
MCU4972307.1	GH4(29-206)	GH4_e30	GH4	alpha-galacturonidase
MCU4972308.1	GH10(8-318)	GH10_e102	GH10	endo-1,4-beta-xylanase
MCU4972327.1	GH42(6-387)	GH42_e1	GH42	beta-galactosidase
MCU4972328.1	PL42(28-333)	PL42_e0	PL42	putative L-rhamnose-α-1,4-D-glucuronate lyase
MCU4972433.1	GH109(3-151)	GH109_e7	-	*N-* acetylgalactosaminidase
MCU4972490.1	GH43_3(46-348)	GH43_e112+CBM13_e196	CBM13+GH43_3	endo-alpha-1,5-L-arabinanase
MCU4972564.1	GH3(90-314)	GH3_e181	GH3	beta-glucosidase/beta-xylosidase
MCU4972716.1	GH5(84-402)	GH5_e92	GH5	endoglucanase
MCU4972717.1	GH5(64-464)	GH5_e92	GH5	endoglucanase
MCU4972718.1	GH5(68-392)	GH5_e92	GH5	endoglucanase
MCU4972720.1	**GH5_7(108-396)**	GH5_e4	**CBM5+GH5_7**	**endo-1,4-beta-mannosidase**
MCU4972721.1	GH5(46-494)	GH5_e197	GH5	endoglucanase
MCU4972722.1	GH10(117-443)	GH10_e16	GH10	endo-1,4-beta-xylanase
MCU4972723.1	GH10(121-461)	GH10_e16	GH10	endo-1,4-beta-xylanase
MCU4972724.1	GH10(118-438)	GH10_e16	GH10	endo-1,4-beta-xylanase
MCU4972725.1	GH5(97-403)	GH5_e92	GH5	endoglucanase
MCU4972728.1	GH5(87-390)	GH5_e92	GH5	endoglucanase
MCU4972729.1	PL14_3(113-318)	PL14_e23	-	alginate PL14
MCU4972730.1	GH5(66-371)	GH5_e197	GH5	endoglucanase
MCU4972733.1	**GH5_7(64-354)**	GH5_e4	**GH5_7**	**endo-1,4-beta-mannosidase**
MCU4972734.1	**GH5_7(92-386)**	GH5_e4	**GH5_7**	**endo-1,4-beta-mannosidase**
MCU4972735.1	GH5(65-356)+CBM6(517-657)	GH5_e197+CBM6_e47	CBM6+GH5	endoglucanase
MCU4972970.1	GH109(3-365)	GH109_e7	-	*N-* acetylgalactosaminidase
MCU4973085.1	GH15(248-609)	GH15_e14	GH15	trehalase
MCU4973255.1	GH93(28-377)	GH93_e13	GH93	alpha-L-arabinofuranobiosidase
MCU4973417.1	GH51(3-499)	GH51_e19	GH51	alpha-L-arabinofuranosidase
MCU4973418.1	GH2(8-471)	GH2_e86	GH2	beta-glucuronidase
MCU4973421.1	GH43_3(32-328)	GH43_e245	GH43_3	endo-alpha-1,5-L-arabinanase
MCU4973427.1	GH2(9-667)	GH2_e86	GH2	beta-galactosidase/beta-glucuronidase
MCU4973431.1	GH127(15-550)	GH127_e0	GH127	beta-L-arabinofuranosidase
MCU4973461.1	GH28(33-390)	GH28_e10	GH28	polygalacturonase
MCU4973474.1	PL1_2(1108-1301)	PL1_e72	PL1	pectate lyase
MCU4973476.1	PL1_2(529-716)	PL1_e72	PL1	pectate lyase
MCU4973506.1	CBM13(565-705)	CBM13_e31	CBM0	mannose-binding CBM
MCU4973507.1	GH2(60-833)	GH2_e101	GH2	beta-galactosidase
MCU4973508.1	GH2(5-482)	GH2_e41	GH2	beta-glucuronidase/beta-galactosidase
MCU4973509.1	PL26(6-867)	PL26_e0	PL26	rhamnogalacturonan lyase
MCU4973513.1	GH28(63-446)	GH28_e139	GH28	polygalacturonase
MCU4973533.1	PL22_2(188-380)	PL22_e5+PL22_e6	PL22	oligogalacturonide lyase
MCU4973534.1	PL22(44-149)+PL22_2(180-374)	PL22_e5+PL22_e6	PL22	oligogalacturonide lyase
MCU4973537.1	CBM67(119-302)+GH78(322-852)	CBM67_e11+GH78_e42	GH78	alpha-L-rhamnosidase
MCU4973540.1	GH51(230-726)	GH51_e48	GH51	alpha-L-arabinofuranosidase
MCU4973541.1	GH106(13-765)	GH106_e6	GH106	alpha-L-rhamnosidase
MCU4973543.1	PL22(46-151)+PL22(192-394)	PL22_e5+PL22_e6	PL22	oligogalacturonide lyase
MCU4973549.1	GH95(7-795)	GH95_e1	GH95	alpha-L-fucosidase
MCU4973550.1	GH43_18(45-275)	GH43_e83	GH43_18	alpha-L-arabinofuranosidase
MCU4973552.1	PL11(1-588)	PL11_e0	PL11_1	rhamnogalacturonan lyase
MCU4973555.1	CBM67(325-482)+GH78(544-1065)	CBM67_e28+GH78_e58	GH78	alpha-L-rhamnosidase
MCU4973556.1	GH2(6-568)	GH2_e144	GH2	beta-glucuronidase
MCU4973558.1	GH2(2-507)	GH2_e145	GH2	beta-galactosidase
MCU4973562.1	GH42(25-404)	GH42_e1	GH42	beta-galactosidase
MCU4973563.1	CBM13(418-510)+CBM13(487-557)	CBM13_e196	CBM13	mannose-binding CBM
MCU4973582.1	PL1_2(109-280)	PL1_e72	PL1	pectate lyase
MCU4973583.1	PL1_2(110-300)	PL1_e72	-	pectate lyase
MCU4973647.1	GH109(6-182)	GH109_e2	-	*N-* acetylgalactosaminidase
MCU4973677.1	GH43_12(5-284)+CBM91(322-526)	GH43_e73+CBM91_e28	GH43_12	alpha-L-arabinofuranosidase
MCU4973710.1	GH9(142-643)	GH9_e1	GH9	endo-beta-1,4_glucanase
MCU4973798.1	CE14(4-113)	CE14_e47	CE14	N-acetyl-alpha-D-glucosaminyl L-malate deacetylase
MCU4973813.1	CE14(17-126)	CE14_e40	-	N-acetyl-alpha-D-glucosaminyl L-malate deacetylase
MCU4973845.1	GH154(11-359)	GH154_e14	GH154	arabinogalactan-beta-1,6-glucuronidase
MCU4973874.1	**CBM88(425-506)**	**CBM88_e1**	**CBM88**	**galactomannan-binding CBM**
MCU4974048.1	GH2(41-943)	GH2_e61	GH2	beta-galactosidase
MCU4974050.1	GH2(36-930)	GH2_e61	GH2	beta-galactosidase
MCU4974053.1	PL42(28-311)	PL42_e11	PL42	putative L-rhamnose-α-1,4-D-glucuronate lyase
MCU4974054.1	GH30_4(135-587)	GH30_e29	GH30_4	endo-beta-1,6-galactanase
MCU4974067.1	GH2(54-645)	GH2_e82	GH2	beta-glucuronidase
MCU4974079.1	GH88(44-378)	GH88_e1	GH88	unsaturated glucuronyl hydrolase
MCU4974103.1	GH95(8-739)	GH95_e1	GH95	alpha-L-fucosidase
MCU4974104.1	GH29(3-348)	GH29_e53	GH29	alpha-L-fucosidase
MCU4974109.1	GH2(5-579)	GH2_e127	GH2	beta-galactosidase/beta-glucuronidase
MCU4974111.1	GH5(59-331)+CBM9(484-651)	GH5_e92+CBM9_e5	CBM9+GH5	endoglucanase
MCU4974196.1	GH2(36-945)	GH2_e92	GH2	beta-galactosidase
MCU4974204.1	CE4(226-344)	CE4_e274	-	xylan deacetylase
MCU4974265.1	GH3(43-254)	GH3_e103	GH3	beta-glucosidase
MCU4974344.1	GH43_12(5-279)+CBM91(315-492)	GH43_e73+CBM91_e27	GH43_12	alpha-L-arabinofuranosidase
MCU4974345.1	GH159(23-239)	GH159_e6	GH159	alpha-arabinofuranosidase/beta-galactofuranosidase
MCU4974390.1	GH4(3-181)	GH4_e21	GH4	alpha-galactosidase
MCU4974704.1	CE4(219-337)	CE4_e274	-	xylan deacetylase
MCU4974899.1	CE15(19-396)	CE15_e20	CE15	4-O-methyl-glucuronoyl methylesterase
MCU4975062.1	GH4(3-181)	GH4_e21	GH4	alpha-galactosidase
MCU4975149.1	GH2(22-579)	GH2_e67	GH0	beta-mannosidase
MCU4975260.1	GH51(290-806)	GH51_e44	GH51	alpha-L-arabinofuranosidase
MCU4975300.1	GH28(29-392)	GH28_e40	GH28	polygalacturonase
MCU4975301.1	PL22(40-152)+PL22_2(203-381)	PL22_e5+PL22_e6	PL22	oligogalacturonate lyase
MCU4975302.1	CBM9(17-222)	CBM9_e3	-	xylan-binding CBM
MCU4975303.1	GH115(21-728)	GH115_e6	GH115	xylan alpha-1,2-glucuronidase
MCU4975305.1	GH4(20-200)	GH4_e30	GH4	alpha-galacturonidase
MCU4975308.1	CBM67(359-507)+GH78(567-1080)	CBM67_e28+GH78_e58	GH78	alpha-L-rhamnosidase
MCU4975309.1	CBM13(589-723)	CBM13_e122	CBM13	mannose-binding CBM
MCU4975450.1	GH88(40-374)	GH88_e1	GH88	unsaturated glucuronyl hydrolase
MCU4975456.1	GH29(2-352)	GH29_e53	GH29	alpha-L-fucosidase
MCU4975457.1	GH137(47-345)	GH137_e1	GH137	beta-L-arabinofuranosidase
MCU4975461.1	GH2(29-582)+GH2(808-1117)	GH2_e1	GH2	beta-galactosidase
MCU4975462.1	GH2(30-696)	GH2_e101	GH2	beta-galactosidase
MCU4975469.1	GH29(3-339)	GH29_e54	-	alpha-L-fucosidase
MCU4975470.1	GH2(31-818)	GH2_e101	GH2	beta-galactosidase
MCU4975481.1	CBM67(114-298)+GH78(320-840)	CBM67_e3+GH78_e32	CBM67+GH78	alpha-L-rhamnosidase
MCU4975482.1	GH106(12-746)	GH106_e6	GH106	alpha-L-rhamnosidase
MCU4975485.1	CBM67(339-504)+GH78(530-1037)	CBM67_e28+GH78_e58	GH78	alpha-L-rhamnosidase
MCU4975491.1	CBM67(141-307)+GH78(331-838)	CBM67_e28+GH78_e58	GH78	alpha-L-rhamnosidase
MCU4975514.1	**GH5_7(89-381)**	**GH5_e239**	**GH5_7**	**endo-1,4-beta-mannosidase**
MCU4975518.1	**GH26(67-364)**	**GH26_e17**	**CBM88+GH26**	**endo-1,4-beta-mannosidase**
MCU4975519.1	GH43_3(44-331)+CBM13(361-502)	GH43_e110+CBM13_e196	CBM13+GH43_3	endo-alpha-1,5-L-arabinanase
MCU4975520.1	**GH5_8(96-291)**	**GH5_e158+CBM5_e73**	**GH5**	**endo-1,4-beta-mannosidase**
MCU4975523.1	**GH2(3-691)**	**GH2_e94**	**GH2**	**beta-mannosidase**
MCU4975525.1	GH109(7-154)	GH109_e7	-	*N-* acetylgalactosaminidase
MCU4975556.1	GH3(86-315)	GH3_e181	GH3	beta-glucosidase/beta-xylosidase
MCU4975693.1	CBM85(71-205)	CBM85_e8	-	CBM
MCU4975761.1	GH4(3-181)	GH4_e21	GH4	alpha-galactosidase
MCU4975789.1	GH95(7-734)	GH95_e1	GH95	alpha-L-fucosidase
MCU4975960.1	GH29(8-315)	GH29_e20	GH29	alpha-L-fucosidase
MCU4975961.1	GH29(4-348)	GH29_e0	GH29	alpha-L-fucosidase
MCU4975962.1	GH29(15-352)	GH29_e73	GH29	alpha-L-fucosidase
MCU4975964.1	GH29(4-360)	GH29_e0	GH29	alpha-L-fucosidase
MCU4976039.1	GH29(5-351)	GH29_e55	-	alpha-L-fucosidase
MCU4976040.1	GH29(3-174)	GH29_e53	-	alpha-L-fucosidase
MCU4976047.1	GH29(15-209)	GH29_e77	-	alpha-L-fucosidase

AArc-xg1-1

Gene ID	HMMER	dbCAN_sub	DIAMOND	Function
MCU4739872	GH15(248-609)	GH15_e14	GH15	trehalase
MCU4740185	GH13_20(292-595)	GH13_e49	GH13	alpha-amylase
MCU4740186	GH15(283-639)	GH15_e46	GH15	putative GH15
MCU4740280	GH105(40-342)	GH105_e35	GH105	unsaturated rhamnogalacturonyl hydrolase
MCU4740338	GH109(5-276)	GH109_e2	-	putative GH109
MCU4740429	GH3(52-260)	GH3_e103	GH3	beta-glucosidase
MCU4740508	GH109(6-273)	GH109_e7	-	*N*- acetylgalactosaminidase
MCU4740618	GH5(65-356)+CBM6(517-657)	GH5_e197+CBM6_e47	CBM6+GH5	endo-beta-1,4-glucanase
MCU4740619	**GH5_7(92-386)**	GH5_e4	**GH5_7**	**endo-1,4-beta-mannosidase**
MCU4740620	**GH5_7(96-387)**	GH5_e4	**GH5_7**	**endo-1,4-beta-mannosidase**
MCU4740623	GH5(66-371)	GH5_e197	GH5	endoglucanase
MCU4740624	PL14_3(113-318)	PL14_e23	-	putative PL14
MCU4740625	GH5(87-390)	GH5_e92	GH5	endo-beta-1,4-glucanase
MCU4740628	GH5(97-403)	GH5_e92	GH5	endo-beta-1,4-glucanase
MCU4740629	GH10(118-438)	GH10_e16	GH10	endo-1,4-beta-xylanase
MCU4740630	GH10(121-457)	GH10_e16	GH10	endo-1,4-beta-xylanase
MCU4740631	GH10(117-443)	GH10_e16	GH10	endo-1,4-beta-xylanase
MCU4740632	GH5(46-494)	GH5_e197	GH5	endo-beta-1,4-glucanase
MCU4740633	GH5_7(108-396)	GH5_e4	CBM5+GH5_7	endo-1,4-beta-mannosidase
MCU4740635	GH5(68-392)	GH5_e92	GH5	endo-beta-1,4-glucanase
MCU4740636	GH5(64-464)	GH5_e92	GH5	endo-beta-1,4-glucanase
MCU4740637	GH5(84-402)	GH5_e92	GH5	endo-beta-1,4-glucanase
MCU4740789	GH3(90-314)	GH3_e181	GH3	beta-glucosidase/beta-xylosidase
MCU4740863	GH43_3(46-348)	GH43_e112+CBM13_e196	CBM13+GH43_3	endo-alpha-1,5-L-arabinanase
MCU4740967	GH9(142-643)	GH9_e1	GH9	endo-beta-1,4-glucanase
MCU4741000	GH43_12(5-284)+CBM91(324-528)	GH43_e73+CBM91_e28	GH43_12	alpha-L-arabinofuranosidase
MCU4741030	GH109(6-182)	GH109_e2	-	*N*- acetylgalactosaminidase
MCU4741094	PL1_2(110-300)	PL1_e72	-	pectate lyase
MCU4741095	PL1_2(109-280)	PL1_e72	PL1	pectate lyase
MCU4741125	GH109(3-151)	GH109_e7	-	*N*- acetylgalactosaminidase
MCU4741257	PL42(28-333)	PL42_e0	PL42	L-rhamnose-alpha-1,4-D-glucuronate lyase
MCU4741258	GH42(6-387)	GH42_e1	GH42	beta-galactosidase
MCU4741283	GH11(19-153)+CBM13(199-287)	GH11_e11+CBM13_e122	-	endo-1,4-beta-xylanase
MCU4741297	CBM85(41-175)	CBM85_e8	-	putative glucomannan-binding CBM
MCU4741532	GH93(28-377)	GH93_e13	GH93	alpha-L-arabinofuranobiosidase
MCU4742000	GH4(3-181)	GH4_e21	GH4	alpha-galactosidase
MCU4742395	PL11(1-588)	PL11_e0	PL11_1	rhamnogalacturonan lyase
MCU4742397	GH43_18(45-275)	GH43_e83	GH43_18	alpha-L-arabinofuranobiosidase
MCU4742398	GH95(7-795)	GH95_e1	GH95	alpha-L-fucosidase
MCU4742404	PL22(44-149)+PL22(190-392)	PL22_e5+PL22_e6	PL22	oligogalacturonide lyase
MCU4742406	GH106(13-773)	GH106_e6	GH106	alpha-L-rhamnosidase
MCU4742407	GH51(229-726)	GH51_e48	GH51	alpha-L-arabinofuranosidase
MCU4742409	-	CBM13_e31	CBM0	putattive mannose-binding CBM
MCU4742410	CBM67(119-302)+GH78(322-852)	CBM67_e11+GH78_e42	GH78	alpha-L-rhamnosidase
MCU4742411	-	CBM13_e123	-	putattive mannose-binding CBM
MCU4742413	PL22(44-149)+PL22_2(180-374)	PL22_e5+PL22_e6	PL22	oligogalacturonide lyase
MCU4742414	PL22_2(188-380)	PL22_e5+PL22_e6	PL22	oligogalacturonide lyase
MCU4742434	GH28(63-446)	GH28_e139	GH28	polygalacturonase
MCU4742438	PL26(6-867)	PL26_e0	PL26	rhamnogalacturonan lyase
MCU4742439	GH2(5-701)	GH2_e41	GH2	beta-galactosidase/beta-glucuronidase
MCU4742440	GH2(60-833)	GH2_e101	GH2	beta-galactosidase
MCU4742441	CBM13(565-705)	CBM13_e31	CBM0	putattive mannose-binding CBM
MCU4742471	PL1_2(529-716)	PL1_e72	PL1	pectate lyase
MCU4742473	PL1_2(909-1102)	PL1_e72	PL1	pectate lyase
MCU4742486	GH28(33-390)	GH28_e10	GH28	polygalacturonase
MCU4742515	GH127(15-550)	GH127_e0	GH127	beta-L-arabinofuranosidase
MCU4742519	GH2(9-665)	GH2_e86	GH2	beta-galactosidase/beta-glucuronidase
MCU4742525	GH43_3(32-328)	GH43_e245	GH43_3	endo-alpha-1,5-L-arabinanase
MCU4742528	GH2(8-471)	GH2_e86	GH2	beta-glucuronidase/beta-galactosidase
MCU4742529	GH51(3-499)	GH51_e19	GH51	alpha-L-arabinofuranosidase
MCU4742569	GH28(29-392)	GH28_e40	GH28	polygalacturonase
MCU4742570	PL22(40-152)+PL22_2(203-381)	PL22_e5+PL22_e6	PL22	oligogalacturonate lyase
MCU4742571	CBM9(17-222)	CBM9_e3	-	xylan-binding CBM
MCU4742572	GH115(20-717)	GH115_e6	GH115	xylan α-1,2-(4-O-methyl)-glucuronidase
MCU4742574	GH4(20-200)	GH4_e30	GH4	alpha-galacturonidase
MCU4742577	CBM67(358-531)+GH78(560-1073)	CBM67_e28+GH78_e58	GH78	alpha-L-rhamnosidase
MCU4742578	CBM13(589-723)	CBM13_e122	CBM13	putattive mannose-binding CBM
MCU4742628	GH2(5-579)	GH2_e127	GH2	beta-galactosidase/beta-glucuronidase
MCU4742630	GH5(59-331)+CBM9(484-651)	GH5_e92+CBM9_e5	CBM9+GH5	endoglucanase
MCU4742717	GH2(36-945)	GH2_e92	GH2	beta-galactosidase
MCU4742725	CE4(226-344)	CE4_e274	-	xylan deacetylase
MCU4742966	CE4(219-337)	CE4_e274	-	xylan deacetylase
MCU4743005	CE14(4-113)	CE14_e47	CE14	N-acetyl-alpha-D-glucosaminyl L-malate deacetylase
MCU4743008	GH109(3-142)	-	-	putative GH109
MCU4743020	CE14(17-126)	CE14_e40	-	N-acetyl-alpha-D-glucosaminyl L-malate deacetylase
MCU4743051	GH32(24-329)	GH32_e80	GH32	sucrose-6-phosphate hydrolase
MCU4743052	GH154(11-359)	GH154_e14	GH154	arabinogalactan beta-1,6-glucuronidase
MCU4743081	CBM88(425-506)	CBM88_e1	CBM88	galactomannan-binding CBM
MCU4743125	GH31(221-716)	GH31_e10	GH31	alpha-xylosidase
MCU4743126	GH5(67-371)	GH5_e197	GH5	endoglucanase
MCU4743128	GH109(22-283)	-	-	putative GH109
MCU4743274	CE14(6-115)	CE14_e40	-	N-acetyl-alpha-D-glucosaminyl L-malate deacetylase
MCU4743280	GH109(5-153)	-	-	putative GH109
MCU4743294	CE15(84-403)+CE15(442-766)	CE15_e17+CE15_e17	CE0	4-O-methyl-glucuronoyl methylesterase
MCU4743296	PL40(597-926)	PL40_e1	-	putative PL40
MCU4743313	CE14(9-118)	CE14_e40	-	diacetylchitobiose deacetylase
MCU4743419	GH109(3-365)	GH109_e7	-	*N*- acetylgalactosaminidase
MCU4743465	PL25(2-133)	PL25_e0	-	ulvan Lyase
MCU4743591	CE15(19-396)	CE15_e20	CE15	4-O-methyl-glucuronoyl methylesterase
MCU4743764	GH3(43-254)	GH3_e103	GH3	beta-glucosidase
MCU4743843	GH43_12(5-279)+CBM91(315-492)	GH43_e73+CBM91_e27	GH43_12	alpha-L-arabinofuranosidase
MCU4743844	GH159(23-239)	GH159_e6	GH159	alpha-arabinofuranosidase/beta-galactofuranosidase
MCU4743889	GH4(3-181)	GH4_e21	GH4	alpha-galactosidase
MCU4743970	GH2(41-943)	GH2_e61	GH2	beta-galactosidase
MCU4743971	GH2(36-930)	GH2_e61	GH2	beta-galactosidase
MCU4743974	PL42(28-311)	PL42_e11	PL42	L-rhamnose-alpha-1,4-D-glucuronate lyase
MCU4743975	GH30_4(135-587)	GH30_e29	GH30_4	endo-beta-1,6-galactanase
MCU4743988	GH2(54-645)	GH2_e82	GH2	beta-glucuronidase
MCU4744001	GH88(44-379)	GH88_e1	GH88	unsaturated glucuronyl hydrolase
MCU4744025	GH95(8-739)	GH95_e1	GH95	alpha-L-fucosidase
MCU4744026	GH29(3-348)	GH29_e0	GH29	alpha-L-fucosidase
MCU4744042	GH3(86-315)	GH3_e181	GH3	beta-glucosidase/beta-xylosidase
MCU4744073	GH109(7-154)	GH109_e7	-	*N*- acetylgalactosaminidase
MCU4744075	**GH2(3-711)**	**GH2_e94**	**GH2**	**beta-mannosidase**
MCU4744078	**GH5_8(96-291)**	**GH5_e158+CBM5_e73**	**GH5**	**endo-1,4-beta-mannosidase**
MCU4744079	GH43_3(44-331)+CBM13(361-502)	GH43_e110+CBM13_e196	CBM13+GH43_3	endo-alpha-1,5-L-arabinanase
MCU4744081	**GH26(67-364)**	**GH26_e17**	**CBM88+GH26**	**endo-1,4-beta-mannosidase**
MCU4744085	**GH5_7(89-381)**	**GH5_e239**	**GH5_7**	**endo-1,4-beta-mannosidase**
MCU4744364	GH29(4-360)	GH29_e0	GH29	alpha-L-fucosidase
MCU4744366	GH29(15-352)	GH29_e73	GH29	alpha-L-fucosidase
MCU4744367	GH29(4-348)	GH29_e0	GH29	alpha-L-fucosidase
MCU4744368	GH29(8-315)	GH29_e20	GH29	alpha-L-fucosidase
MCU4744628	CBM67(325-482)+GH78(544-1065)	CBM67_e28+GH78_e58	GH78	alpha-L-rhamnosidase
MCU4744629	GH2(6-568)	GH2_e144	GH2	beta-glucuronidase
MCU4744631	GH2(2-507)	GH2_e145	GH2	beta-galactosidase
MCU4744635	GH42(25-404)	GH42_e1	GH42	beta-galactosidase
MCU4744636	CBM13(418-510)	CBM13_e196	CBM13	mannose-binding CBM
MCU4744644	GH29(3-373)	GH29_e0	GH29	alpha-L-fucosidase
MCU4744645	GH29(5-351)	GH29_e55	-	alpha-L-fucosidase
MCU4744664	GH10(8-318)	GH10_e102	GH10	endo-1,4-beta-xylanase
MCU4744665	GH4(29-206)	GH4_e30	GH4	alpha-galacturonidase
MCU4744667	GH3(66-292)	GH3_e88	GH3	beta-xylosidase/beta-glucosidase
MCU4744674	GH67(8-688)	GH67_e1	GH67	alpha-1,2-glucuronosidase
MCU4744675	CBM85(106-238)+CBM85(329-460)+GH10(518-835)	CBM85_e8+CBM85_e8+GH10_e16	CBM85+GH10	endo-1,4-beta-xylanase
MCU4744676	CBM6(2-87)	CBM6_e65	-	cellulose/xylan-binding CBM
MCU4744742	GH29(3-299)	GH29_e0	GH29	alpha-L-fucosidase
MCU4744746	GH95(7-734)	GH95_e1	GH95	alpha-L-fucosidase
MCU4744766	GH51(289-807)	GH51_e44	GH51	alpha-1,5-L-arabinofuranosidase

KZCA124

Gene ID	HMMER	dbCAN_sub	DIAMOND	Function
WP_207586340	GH43_12(5-298)+CBM91(334-512)	GH43_e73+CBM91_e27	GH43_12	alpha-L-arabinofuranosidase
WP_207586391	GH159(36-252)	GH159_e6	GH159	alpha-arabinofuranosidase/ beta-D-galactofuranosidase
WP_207586522	GH5(66-369)	GH5_e197	GH5	endo-1,4-beta-glucanase
WP_207586523	GH31(221-716)	GH31_e10	GH31	alpha-xylosidase
WP_207586533	GH10(8-321)	GH10_e102	GH10	endo-1,4-beta-xylanase
WP_207586534	GH4(30-207)	GH4_e30	GH4	alpha-galacturonidase
WP_207586542	CBM85(106-240)+CBM85(329-461)+GH10(521-835)	CBM85_e8+CBM85_e8+GH10_e16	CBM85+GH10	endo-1,4-beta-xylanase
WP_207586566	PL42(28-333)	PL42_e0	PL42	L-rhamnose-alpha-1,4-D-glucuronate lyase
WP_207586805	GH9(112-611)	GH9_e1	GH9	endo-1,4-beta-glucanase
WP_207586872	GH3(86-315)	GH3_e181	GH3	beta-glucosidase/beta-xylosidase
WP_207586899	GH109(7-154)	GH109_e7	-	*N*-acetylgalactosaminidase
WP_207586901	**GH2(3-715)**	**GH2_e94**	**GH2**	**beta-mannosidase**
WP_207586903	**GH26(50-339)**	**GH26_e17**	**CBM88+GH26**	**endo-1,4-beta-mannosidase**
WP_207586907	**GH5_7(88-380)**	**GH5_e239**	**GH5_7**	**endo-1,4-beta-mannosidase**
WP_207586908	**GH5_7(91-372)**	**GH5_e4**	**GH5_7**	**endo-1,4-beta-mannosidase**
WP_207587007	CE4(224-341)	CE4_e274	-	putative CE4
WP_207587897	GH105(40-342)	GH105_e35	GH105	unsaturated rhamnogalacturonyl hydrolase
WP_207588032	CE19(42-387)	CE19_e4	-	pectin methylesterase
WP_207588272	GH29(3-373)	GH29_e0	GH29	alpha-L-fucosidase
WP_207588292	GH15(292-649)	GH15_e46	GH15	putative GH15
WP_207588294	GH13(301-612)	GH13_e49	GH13	alpha-amylase
WP_207588358	GH3(51-259)	GH3_e103	GH3	beta-glucosidase
WP_207589397	GH4(3-181)	GH4_e21	GH4	alpha-galactosidase
WP_207589594	CE15(21-398)	CE15_e20	CE15	4-O-methyl-glucuronoyl methylesterase
WP_207589600	PL14(98-307)	PL14_e16	-	alginate lyase
WP_207589934	GH29(5-351)	GH29_e55	-	alpha-L-fucosidase
WP_207589936	GH29(3-373)	GH29_e0	GH29	alpha-L-fucosidase
WP_207589951	GH29(5-360)	GH29_e0	GH29	alpha-L-fucosidase
WP_207589953	GH29(15-352)	GH29_e73	GH29	alpha-L-fucosidase
WP_207589955	GH29(5-349)	GH29_e0	GH29	alpha-L-fucosidase
WP_207589957	GH29(7-315)	GH29_e20	GH29	alpha-L-fucosidase
WP_207589979	GH39(12-462)	GH39_e9	GH39	alpha-L-iduronidase
WP_207590926	GH10(92-424)	GH10_e16	GH10	endo-1,4-beta-xylanase
WP_207591059	GH3(90-314)	GH3_e181	GH3	beta-glucosidase/beta-xylosidase
WP_207591162	GH172(88-338)	GH172_e0	GH172	difructose dianhydride I synthase/hydrolase
WP_207591304	GH5(68-409)	GH5_e197	GH5	endo-beta-1,4-glucanase
WP_207591306	**GH5_7(97-385)**	**GH5_e4**	**CBM5+GH5_7**	**endo-1,4-beta-mannosidase**
WP_207591307	GH5(50-499)	GH5_e197	GH5	endoglucanase
WP_207591308	GH10(119-455)	GH10_e16	GH10	endo-1,4-beta-xylanase
WP_207591309	GH5(99-405)	GH5_e92	GH5	endo-beta-1,4-glucanase
WP_207591312	GH81(98-741)	GH81_e12	GH81	endo-1,3-beta-glucanase
WP_207591313	GH5(90-391)	GH5_e92	GH5	endo-beta-1,4-glucanase
WP_207591314	GH5(66-370)	GH5_e197	GH5	endo-beta-1,4-glucanase
WP_207591317	**GH5_7(92-381)**	**GH5_e4**	**GH5_7**	**endo-1,4-beta-mannosidase**
WP_207591319	**GH5_7(92-385)**	**GH5_e4**	**GH5_7**	**endo-1,4-beta-mannosidase**
WP_207591322	GH5(64-356)+CBM6(516-654)	GH5_e197+CBM6_e47	CBM6+GH5	endo-beta-1,4-glucanase
WP_207591513	GH3(43-254)	GH3_e103	GH3	beta-glucosidase
WP_207591535	CE12(192-378)	CE12_e31	CE12	rhamnogalacturonan acetylesterase
WP_207591776	GH2(7-530)	GH2_e59	GH2	beta-glucuronidase
WP_207591839	CE6(121-225)+CBM88(306-395)	CE6_e3	CBM5+CE6	carbohydrate acetyl esterase/feruloyl esterase
WP_207591864	GH4(3-181)	GH4_e21	GH4	alpha-galactosidase
WP_207592203	CBM13(424-566)	CBM13_e196	CBM13	mannose-binding CBM
WP_207592224	GH51(5-492)	GH51_e19	GH51	alpha-L-arabinofuranosidase
WP_207592240	GH154(11-359)	GH154_e14	GH154	beta-1,6-D-glucuronidase
WP_207592241	GH32(24-318)	GH32_e85	GH32	sucrose-6-phosphate hydrolase
WP_207592273	PL33(411-552)	PL33_e14	PL33_2	hyaluronate lyase
WP_207592299	CE14(17-126)	CE14_e40	-	chitin deacetylase
WP_207592322	GH51(3-500)	GH51_e19	GH51	alpha-L-arabinofuranosidase
WP_207592323	GH2(8-471)	GH2_e86	GH2	beta-galactosidase/beta-glucuronidase
WP_207592325	GH51(256-717)	GH51_e48	GH51	alpha-L-arabinofuranosidase
WP_207592326	GH43_3(3-293)	GH43_e245	GH43_3	endo-alpha-1,5-L-arabinanase
WP_207592331	GH2(7-693)	GH2_e86	GH2	beta-galactosidase/beta-glucuronidase
WP_207592335	GH127(15-550)	GH127_e0	GH127	beta-L-arabinofuranosidase
WP_207592356	PL1_2(432-615)	PL1_e72	PL1	pectate lyase
WP_207592357	GH28(33-389)	GH28_e10	GH28	polygalacturonase
WP_207592371	PL1_2(77-261)+PL1_2(568-745)	PL1_e72+PL1_e72	PL1	pectate lyase
WP_207592372	PL1_2(477-655)+PL1_2(969-1153)	PL1_e72+PL1_e72	PL1	pectate lyase
WP_207592406	GH2(8-558)	GH2_e59	GH2	beta-glucuronidase
WP_207592426	GH51(296-812)	GH51_e44	GH51	alpha-1,5-L-arabinofuranosidase
WP_207592460	GH28(28-390)	GH28_e40	GH28	polygalacturonase
WP_207592461	PL22(30-152)+PL22_2(195-373)	PL22_e5+PL22_e6	PL22	oligogalacturonide lyase
WP_207592462	CBM9(17-222)	-	-	xylan-binding CBM
WP_207592463	GH115(21-665)	GH115_e6	GH115	xylan alpha-1,2-glucuronidase
WP_207592465	GH4(20-200)	GH4_e30	GH4	alpha-galacturonidase
WP_207592468	CBM67(367-519)+GH78(579-1087)	CBM67_e28+GH78_e58	GH78	alpha-L-rhamnosidase
WP_207592469	CBM13(595-729)	CBM13_e122	CBM13	mannose-binding CBM
WP_207592492	GH38(274-528)	**GH38_e18**	**GH38**	**alpha-mannosidase**
WP_207592514	GH2(6-561)	GH2_e127	GH2	beta-galactosidase/beta-glucuronidase
WP_207592519	GH42(6-392)	GH42_e1	GH42	beta-galactosidase
WP_207592521	GH5(47-322)+CBM9(469-641)	GH5_e92+CBM9_e5	CBM9+GH5	endo-beta-1,4-glucanase
WP_207592615	CE12(7-214)	CE12_e12	CE12	rhamnogalacturonan acetylesterase
WP_207592617	CBM13(394-509)	CBM13_e196	CBM13	mannose-binding CBM
WP_207592618	GH42(6-385)	GH42_e1	GH42	beta-galactosidase
WP_207592619	GH43_3(47-336)	GH43_e110	CBM13+GH43_3	endo-alpha-1,5-L-arabinanase
WP_207592621	GH2(2-505)	GH2_e145	GH2	beta-galactosidase
WP_207592624	PL26(279-786)	PL26_e0	-	rhamnogalacturonan lyase
WP_207592625	GH2(6-579)	GH2_e144	GH2	beta-glucuronidase
WP_207592626	CBM67(331-481)+GH78(545-1068)	CBM67_e28+GH78_e58	GH78	alpha-L-rhamnosidase
WP_207592631	CBM13(552-683)	CBM13_e294	CBM13	mannose-binding CBM
WP_207592634	PL22(45-144)	PL22_e5+PL22_e6	PL22	oligogalacturonate lyase
WP_207592635	GH43_18(43-277)	GH43_e83	GH43_18	alpha-L-arabinofuranosidase
WP_207592636	GH95(7-790)	GH95_e1	GH95	alpha-L-fucosidase
WP_207592640	PL22(40-146)+PL22(188-391)	PL22_e5+PL22_e6	PL22	oligogalacturonide lyase
WP_207592643	PL1_2(483-663)	PL1_e72	PL1	pectate lyase
WP_207592645	GH106(13-774)	GH106_e6	GH106	alpha-L-rhamnosidase
WP_207592646	GH51(226-721)	GH51_e48	GH51	alpha-L-arabinofuranosidase
WP_207592648	-	CBM13_e294	CBM0	mannose-binding CBM
WP_207592649	CBM67(119-313)+GH78(333-863)	CBM67_e11+GH78_e42	GH78	alpha-L-rhamnosidase
WP_207592650	CBM13(165-303)	CBM13_e294	-	mannose-binding CBM
WP_207592651	PL22(45-149)+PL22_2(180-375)	PL22_e5+PL22_e6	PL22	oligogalacturonide lyase
WP_207592652	PL22_2(189-381)	PL22_e6	PL22	oligogalacturonide lyase
WP_207592672	GH28(69-445)	GH28_e139	GH28	polygalacturonase
WP_207592674	CE19(36-331)	CE19_e1	-	pectin methylesterase
WP_207592703	GH88(43-378)	GH88_e1	GH88	unsaturated glucuronyl hydrolase
WP_207592754	GH3(76-303)	GH3_e204	GH3	beta-hexosaminidase
WP_207592758	GH20(150-475)	GH20_e51	GH20	beta-hexosaminidase
WP_207592761	CE14(6-115)	CE14_e40	-	pectin methylesterase
WP_207592779	CE15(83-403)+CE15(442-767)	CE15_e17+CE15_e17	CE0	4-O-methyl-glucuronoyl methylesterase
WP_207592791	PL40(597-925)	PL40_e1	PL0	putative PL40
WP_207592810	CE14(9-118)	CE14_e40	-	diacetylchitobiose deacetylase
WP_207592899	GH154(11-351)	GH154_e14	GH154	arabinogalactan beta-1,6-glucuronidase
WP_207592907	GH2(6-562)	GH2_e144	GH2	beta-glucuronidase
WP_207592910	PL26(6-869)	PL26_e0	PL26	rhamnogalacturonan lyase
WP_207592913	GH2(7-489)	GH2_e41	GH2	beta-galactosidase/beta-glucuronidase
WP_207592948	GH97(3-591)	GH97_e29	GH97	alpha-galactosidase
WP_207592950	CE14(8-117)	CE14_e40	-	diacetylchitobiose deacetylase
WP_207593013	PL11(1-591)	PL11_e0	PL11_1	rhamnogalacturonan lyase
WP_242695398	GH3(93-319)	GH3_e88	GH3	beta-xylosidase/beta-glucosidase
WP_242695399	GH67(8-688)	GH67_e1	GH67	alpha-1,2-glucuronosidase
WP_242695417	GH27(109-371)	GH27_e14	GH27	alpha-galactosidase
WP_242695420	**GH5_8(96-291)**	**GH5_e158+CBM5_e73**	**GH5**	**endo-1,4-beta-mannosidase**
WP_242695508	PL14(114-319)	PL14_e16	-	alginate lyase
WP_242695510	PL14(56-266)	PL14_e16	-	alginate lyase
WP_242695594	PL11(1-576)	PL11_e0	CBM13+PL11_1	rhamnogalacturonan lyase
WP_242695613	PL1_2(380-548)	PL1_e72	PL1	pectate lyase
WP_242695657	GH3(91-317)	GH3_e88	GH3	beta-xylosidase/beta-glucosidase
WP_255542848	GH5(61-465)	GH5_e92	GH5	endo-1,4-beta-glucanase
WP_255543154	GH20(3-242)	GH20_e78	GH20	beta-hexosaminidase
WP_305037870	GH11(78-251)+CBM13(279-415)	GH11_e15+CBM13_e122	CBM13+GH11	endo-1,4-beta-xylanase

TS33

Gene ID	HMMER	dbCAN_sub	DIAMOND	Function
WP_276252459.1	GH5(64-356)+CBM6(516-654)	GH5_e197+CBM6_e47	CBM6+GH5	endo-1,4-beta-glucanase
WP_276252460.1	**GH5_7(92-385)**	**GH5_e4**	**GH5_7**	**endo-1,4-beta-mannosidase**
WP_276252461.1	**GH5_7(92-381)**	**GH5_e4**	**GH5_7**	**endo-1,4-beta-mannosidase**
WP_276252462.1	-	-	CBM5	cellulose-binding CBM
WP_276252464.1	GH5(66-370)	GH5_e197	GH5	endo-1,4-beta-glucanase
WP_276252465.1	GH5(91-392)	GH5_e92	GH5	endo-1,4-beta-glucanase
WP_276252467.1	GH5(109-415)	GH5_e92	GH5	endo-1,4-beta-glucanase
WP_276252468.1	GH10(113-449)	GH10_e16	GH10	endo-1,4-beta-xylanase
WP_276252469.1	GH5(49-497)	GH5_e197	GH5	endo-1,4-beta-glucanase
WP_276252470.1	**GH5_7(97-385)**	**GH5_e4**	**CBM5+GH5_7**	**endo-1,4-beta-mannosidase**
WP_276252472.1	GH5(68-409)	GH5_e197	GH5	endoglucanase
WP_276252473.1	GH5(61-465)	GH5_e92	GH5	endoglucanase
WP_276252597.1	GH3(90-314)	GH3_e181	GH3	beta-glucosidase/beta-xylosidase
WP_276252658.1	GH10(3-213)	GH10_e16	-	endo-1,4-beta-xylanase
WP_276252661.1	CBM57(88-224)	CBM57_e32	-	broad-specificity CBM
WP_276252663.1	GH10(50-367)	GH10_e16	GH10	endo-1,4-beta-xylanase
WP_276252664.1	GH43_3(46-348)+CBM13(374-513)	GH43_e112+CBM13_e196	CBM13+GH43_3	endo-alpha-1,5-L-arabinanase
WP_276252754.1	GH2(22-582)	GH2_e67	GH0	beta-galactosidase
WP_276253105.1	GH95(6-734)	GH95_e1	GH95	alpha-L-fucosidase
WP_276253111.1	GH29(3-369)	GH29_e0	GH29	alpha-L-fucosidase
WP_276253115.1	GH95(11-738)	GH95_e1	GH95	alpha-L-fucosidase
WP_276253116.1	PL1_2(90-260)	PL1_e72	PL1	pectate lyase
WP_276253173.1	PL12(345-479)	PL12_e12	PL0	heparin-sulfate lyase
WP_276253290.1	CBM6(208-343)	CBM6_e4	-	xylan-binding CBM
WP_276253291.1	PL14(156-358)	PL14_e16	-	alginate lyase
WP_276253293.1	PL14(25-229)	PL14_e16	-	alginate lyase
WP_276253309.1	CE15(19-396)	CE15_e20	CE15	4-O-methyl-glucuronoyl methylesterase
WP_276253409.1	GH4(3-181)	GH4_e21	GH4	alpha-galactosidase
WP_276253872.1	GH3(55-263)	GH3_e103	GH3	beta-glucosidase
WP_276253901.1	GH13(301-614)	GH13_e49	GH13	alpha-amylase
WP_276253902.1	GH15(292-649)	GH15_e46	GH15	alpha-amylase
WP_276253913.1	GH29(3-373)	GH29_e0	GH29	alpha-L-fucosidase
WP_276254039.1	CE19(42-387)	CE19_e4	-	pectin methylesterase
WP_276254127.1	GH105(40-342)	GH105_e35	GH105	unsaturated rhamnogalacturonyl hydrolase
WP_276254560.1	CE4(224-341)	CE4_e274	-	chtinin deacetylase
WP_276254647.1	**GH5_7(91-372)**	**GH5_e4**	**GH5_7**	**endo-1,4-beta-mannosidase**
WP_276254648.1	**GH5_7(88-380)**	**GH5_e239**	**GH5_7**	**endo-1,4-beta-mannosidase**
WP_276254652.1	**GH26(50-339)**	**GH26_e17**	**CBM88+GH26**	**endo-1,4-beta-mannosidase**
WP_276254653.1	**GH5_8(96-291)**	**GH5_e158+CBM5_e73**	**GH5**	**endo-1,4-beta-mannosidase**
WP_276254656.1	**GH2(3-706)**	**GH2_e94**	**GH2**	**beta-mannosidase**
WP_276254678.1	GH27(96-358)	GH27_e14	GH27	alpha-galactosidase
WP_276254680.1	GH3(86-315)	GH3_e181	GH3	beta-glucosidase/beta-xylosidase
WP_276254736.1	GH9(112-611)	GH9_e1	GH9	endo-1,4-beta-glucanase
WP_276254969.1	PL42(28-333)	PL42_e0	PL42	rhamnose-alpha-1,4-gucuronate lyase
WP_276254993.1	GH11(62-235)+CBM13(262-398)	GH11_e15+CBM13_e122	CBM13+GH11	endo-1,4-beta-xylanase
WP_276254994.1	CBM85(106-240)+CBM85(329-461)+GH10(520-834)	CBM85_e8+CBM85_e8+GH10_e16	CBM85+GH10	endo-1,4-beta-xylanase
WP_276254995.1	GH67(8-688)	GH67_e1	GH67	alpha-1,2-glucuronosidase
WP_276255002.1	GH3(93-319)	GH3_e88	GH3	beta-xylosidase/beta-glucosidase
WP_276255004.1	GH4(30-207)	GH4_e30	GH4	alpha-galacturonidase
WP_276255006.1	GH10(8-321)	GH10_e102	GH10	endo-1,4-beta-xylanase
WP_276255016.1	GH31(221-716)	GH31_e10	GH31	alpha-xylosidase
WP_276255017.1	GH5(66-369)	GH5_e197	GH5	endo-1,4-beta-glucanase
WP_276255134.1	GH159(36-252)	GH159_e6	GH159	alpha-arabinofuranosidase/beta-galactofuranosidase
WP_276255183.1	GH43_14(3-298)+CBM91(334-512)	GH43_e73+CBM91_e27	GH43_12	alpha-L-arabinofuranosidase
WP_276255396.1	GH4(3-181)	GH4_e21	GH4	alpha-galactosidase
WP_276255415.1	CE6(121-225)+CBM88(306-395)	CE6_e3	CBM5+CE6	carbohydrate acetyl esterase/feruloyl esterase
WP_276255588.1	CE12(192-378)	CE12_e31	CE12	rhamnogalacturonan acetylesterase
WP_276255624.1	GH3(43-254)	GH3_e103	GH3	beta-glucosidase
WP_276255695.1	PL11(1-575)	PL11_e0	CBM13+PL11_1	rhamnogalacturonan lyase
WP_276255813.1	GH88(43-378)	GH88_e1	GH88	unsaturated glucuronyl hydrolase
WP_276255844.1	CE19(36-331)	CE19_e1	-	pectin methylesterase

WP_276255845.1 GH28(69-445)	GH28_e139	GH28	polygalacturonase
WP_276255863.1 PL22_2(189-381)	PL22_e6	PL22	oligogalacturonide lyase
WP_276255864.1 PL22(45-149)+PL22_2(180-374)	PL22_e5+PL22_e6	PL22	oligogalacturonide lyase
WP_276255866.1 CBM13(165-303)	CBM13_e294	-	mannose-binding CBM
WP_276255867.1 CBM67(119-313)+GH78(333-863)	CBM67_e11+GH78_e42	GH78	alpha-L-rhamnosidase
WP_276255870.1 GH51(226-720)	GH51_e48	GH51	alpha-L-arabinofuranosidase
WP_276255871.1 GH106(13-774)	GH106_e6	GH106	alpha-L-rhamnosidase
WP_276255873.1 PL1_2(483-665)	PL1_e72	PL1	pectate lyase
WP_276255876.1 PL22(48-154)+PL22(196-399)	PL22_e5+PL22_e6	PL22	oligogalacturonide lyase
WP_276255880.1 GH95(7-797)	GH95_e1	GH95	alpha-L-fucosidase
WP_276255881.1 GH43_18(43-277)	GH43_e83	GH43_18	alpha-L-arabinofuranosidase
WP_276255882.1 PL22(45-144)	PL22_e5+PL22_e6	PL22	oligogalacturonide lyase
WP_276255884.1 CBM13(552-683)	CBM13_e294	CBM13	mannose-binding CBM
WP_276255888.1 CBM67(332-481)+GH78(545-1068)	CBM67_e28+GH78_e58	GH78	alpha-L-rhamnosidase
WP_276255889.1 GH2(6-579)	GH2_e144	GH2	beta-glucuronidase/beta-galactosidase
WP_276255890.1 PL26(279-786)	PL26_e0	-	rhamnogalacturonan lyase
WP_276255893.1 GH2(2-740)	GH2_e145	GH2	beta-galactosidase
WP_276255895.1 GH43_3(47-334)	GH43_e110	CBM13+GH43_3	endo-alpha-1,5-L-arabinanase
WP_276255896.1 GH42(6-385)	GH42_e1	GH42	beta-galactosidase
WP_276255897.1 CBM13(398-509)+CBM13(487-557)	CBM13_e196	CBM13	mannose-binding CBM
WP_276255899.1 CE12(7-214)	CE12_e12	CE12	rhamnogalacturonan acetylesterase
WP_276255906.1 GH2(8-558)	GH2_e59	GH2	beta-glucuronidase
WP_276255944.1 PL1_2(77-261)+PL1_2(563-741)+PL1_2(1055-1239)	PL1_e72+PL1_e72+PL1_e72	PL1	pectate lyase
WP_276255959.1 GH28(33-389)	GH28_e10	GH28	polygalacturonase
WP_276255960.1 PL1_2(432-615)	PL1_e72	PL1	pectate lyase
WP_276255991.1 GH127(15-550)	GH127_e0	GH127	beta-L-arabinofuranosidase
WP_276255992.1 GH2(4-691)	GH2_e86	GH2	beta-galactosidase/beta-glucuronidase
WP_276255998.1 GH51(3-504)	GH51_e19	GH51	alpha-L-arabinofuranosidase
WP_276256021.1 CE14(17-126)	CE14_e40	-	diacetylchitobiose deacetylase
WP_276256033.1 -	-	CE0	putative CE
WP_276256048.1 PL33(411-552)	PL33_e14	PL33_2	putative PL33
WP_276256079.1 GH32(24-322)	GH32_e85	GH32	sucrose-6-phosphate hydrolase
WP_276256080.1 GH154(11-359)	GH154_e14	GH154	beta-1,6-D-glucuronidase
WP_276256114.1 CBM13(424-566)	CBM13_e196	CBM13	mannose-binding CBM
WP_276256221.1 CE14(8-117)	CE14_e40	-	diacetylchitobiose deacetylase
WP_276256223.1 GH13_31(33-394)	GH13_e122	GH13_31	oligo-1,6-glucosidase
WP_276256226.1 GH97(3-592)	GH97_e8	GH97	alpha-galactosidase
WP_276256271.1 CBM13(173-313)	CBM13_e122	-	mannose-binding CBM
WP_276256272.1 GH2(61-833)	GH2_e101	GH2	beta-galactosidase
WP_276256273.1 GH2(7-482)	GH2_e41	GH2	beta-glucuronidase/beta-galactosidase
WP_276256275.1 GH2(6-565)	GH2_e144	GH2	beta-glucuronidase
WP_276256284.1 GH154(11-351)	GH154_e14	GH154	beta-1,6-D-glucuronidase
WP_276256370.1 CE15(84-403)+CE15(442-777)	CE15_e17+CE15_e17	CE0	4-O-methyl-glucuronoyl methylesterase
WP_276256392.1 CE14(6-115)	CE14_e40	-	chitin deacetylase
WP_276256395.1 GH20(147-472)	GH20_e51	GH20	beta-hexosaminidase
WP_276256399.1 GH3(77-303)	GH3_e204	GH3	beta-hexosaminidase
WP_276256401.1 GH20(152-477)	GH20_e51	GH20	beta-hexosaminidase
WP_276256402.1 -	-	PL0	putative PL
WP_276256420.1 GH20(129-442)	GH20_e75	GH20	beta-hexosaminidase
WP_276256459.1 CE14(9-118)	CE14_e40	-	chitin deacetylase
WP_276256465.1 PL11(1-591)	PL11_e0	PL11_1	rhamnogalacturonan lyase
WP_276256467.1 PL1_2(386-554)	PL1_e72	PL1	putative PL1
WP_276256582.1 GH97(3-580)	GH97_e29	GH97	alpha-galactosidase
WP_276256591.1 GH97(28-680)	GH97_e24	GH97	alpha-glucosidase
WP_276256607.1 GH42(8-398)+GH164(304-656)	GH42_e24+GH164_e0	GH42	beta-galactosidase
WP_276256616.1 GH99(3-289)	GH99_e4	-	endo-alpha-1,2-mannosidase
WP_276256619.1 GH38(97-374)	GH38_e13	GH38	alpha-mannosidase
WP_276256621.1 GH38(7-289)	GH38_e33	GH38	alpha-mannosidase
WP_276256622.1 -	-	GH38	alpha-mannosidase
WP_276256625.1 GH141(15-547)	GH141_e24	GH141	alpha-L-fucosidase/xylanase
WP_276256649.1 GH33(17-265)	GH33_e136	GH0	putative GH33
WP_276256653.1 **GH2(17-759)**	**GH2_e94**	**GH2**	**beta-mannosidase**
WP_276256656.1 GH38(274-527)	GH38_e38	GH38	alpha-mannosidase

WP_276256657.1	-	-	GH38	alpha-mannosidase
WP_276256658.1	GH38(7-289)	GH38_e33	GH38	alpha-mannosidase
WP_276256659.1	GH29(22-369)	GH29_e83+CBM32_e149	CBM32+GH29	alpha-L-fucosidase
WP_276256660.1	GH29(3-353)	GH29_e0	GH29	alpha-L-fucosidase
WP_276256663.1	GH20(130-383)	GH20_e75	GH20	beta-hexosaminidase
WP_276256664.1	GH29(4-351)	GH29_e54	GH29	alpha-L-fucosidase
WP_276256675.1	GH18(20-316)	GH18_e66	GH18	endo-chitinase
WP_276256688.1	GH33(303-595)	GH33_e4	GH33	putative GH33
WP_276256692.1	CBM9(16-212)	-	-	xylan-binding CBM
WP_276256702.1	GH171(23-388)	GH171_e0	GH171	beta-N-acetylmuramidase
WP_276256708.1	GH171(22-388)	GH171_e0	GH171	beta-N-acetylmuramidase
WP_276256822.1	GH3(80-303)	GH3_e1	GH3	beta-glucosidase/beta-xylosidase
WP_276256823.1	GH144(45-442)	GH144_e3	GH144	endo-beta-1,2-glucanase
WP_276256825.1	GH172(87-329)	GH172_e0	GH172	difructose dianhydride I synthase/hydrolase
WP_276256828.1	CBM67(6-174)+GH78(237-734)	CBM67_e16+GH78_e57	CBM67+GH78	alpha-L-rhamnosidase
WP_276256835.1	-	CBM13_e294	CBM0+PL11_1	rhamnogalactutonan lyase
WP_276256844.1	CBM67(363-516)+GH78(576-1084)	CBM67_e28+GH78_e58	GH78	alpha-L-rhamnosidase
WP_276256847.1	GH4(20-200)	GH4_e30	GH4	alpha-galacturonidase
WP_276256849.1	GH115(21-970)	GH115_e6	GH115	xylan alpha-1,2-glucuronidase
WP_276256850.1	CBM9(17-222)	-	-	xylan-binding CBM
WP_276256851.1	PL22(30-153)+PL22_2(198-376)	PL22_e5+PL22_e6	PL22	oligogalacturonate lyase
WP_276256852.1	GH28(28-390)	GH28_e40	GH28	polygalacturonase
WP_276256884.1	GH51(5-484)	GH51_e44	GH51	alpha-L-arabinofuranosidase
WP_276257005.1	CE4(221-340)	CE4_e274	-	chitin deacetylase CE4
WP_276257014.1	GH2(36-940)	GH2_e61	GH2	beta-galactosidase
WP_276257091.1	GH5(47-315)+CBM9(469-641)	GH5_e92+CBM9_e5	CBM9+GH5	endoglucanase
WP_276257093.1	GH42(6-392)	GH42_e1	GH42	beta-galactosidase
WP_276257099.1	GH2(6-580)	GH2_e127	GH2	beta-galactosidase/beta-glucuronidase
WP_276257120.1	GH38(274-528)	GH38_e18	GH38	alpha-mannosidase
WP_276257139.1	CBM13(231-366)	CBM13_e31	CBM13	mannose-binding CBM

Table S5.

Protein subunits	Protein name/function	Locus tag (MCU497+)
**Ion/pH homeastasis**		
TrkAH locus	Trk system: K^+/H^+ symporter	4800-4809
TrkA_C-terminal	Potassium import : single subunits	2522-2523; 3340;5908
TrkA_N-terminal		2524; 2622; 3163
KdpAB/<br>TrkAH	High-affinity K^+-uptake ATPase/<br>Potassium symporter	5901-5902/<br>5903-5904
CPA2	K^+/H^+ antiporter	1449; 1718
KefB	K^+/H^+ antiporter (efflux)	1717
Kch<br>PchB<br>Two-pore channel	Potassium channels	1397<br>3702<br>1167
NatAB1B2	Na^+-efflux ABC transporter	1569-1561
**Ca/Mg uptake**		
CaCA	Ca^+/Na^+ antiporter	2699; 3161; 5166
MtgE	Magnesium transporter	1398-1399; 3648-3649
**Na^+/H^+ antiporters**		
NhaP	Single subunit Na(K) $^+/H^+$ antiporter	1720; 4800
MrpB1B2CD1D2D3EFG	multisubuinit Na^+/H^+ antiporter	4115-4123
**Oxidative stress response components**		
KatG	Catalase/peroxidase HPI	1889
KatE	Catalase	5270
AhpD	Alkylperoxidase	1633
Bcp	PeroxiredoxinQ	1964
Sod	[Mn] superoxide dismutase	1592; 5621
**Respiratory cytochrome complexes**		
heme-copper cytochrome *c* oxidase aa_3		
CoxD/B/AC	Catalytic subunit IV/II/I-III	3230/3253/3302
cytochrome *c*/quinol oxidase ba_3		
CbaA1B/CbdA/TlpA/CSO	Subunit I-II/cytochrome c biogenesis	5683-5687
CbaEDA2C	Catalytic subunits III-V	5809-5812
CtaB/Cox10	Heme *o* synthase	3407; 5690
CtaA/Cox15	Heme *a* synthase	1395
**NADH-MK oxido-reductase**		
PetABDE	Haloarchaeal complex III	4564-4568
**Aerobic type of CO-dehydrogenase**		
CoxSML/I	Mo-Cu CO dehydrogenase	1179-1182
**Denitrification**		
HpcE/ NirK	Plastocyanin/Cu-nitrite reductase	5688-5689
NorZ (qNorB)	Archaeal NO-reductase	5222
NosL1Y1F1DL2/Z	N_2O-reductase operon1	3623-3628
NosF2Y2L3	N_2O-reductase operon2	5815-5817
**Periplasmic halocyanin/plastocyanin Cu-proteins** (replacements for cytochrome *c*)		
Hcy	Halocyanin family	1867; 2083; 3906
HcpE	Plastocyanin/azurin family	1806; 2124-2126;<br>3339; 4564; 4612
GlbN	Cyanoglobin (truncated hemoglobin)	2335
UrtABCD<br>UreABC<br>UreDEFG	Urea ABC transporter<br>Urease catalytic subunits<br>Urease accessory proteins	3492-3495<br>3496-3498<br>3498-34502
PhaCEP	Archaeal PHA synthase type III	5584-5586

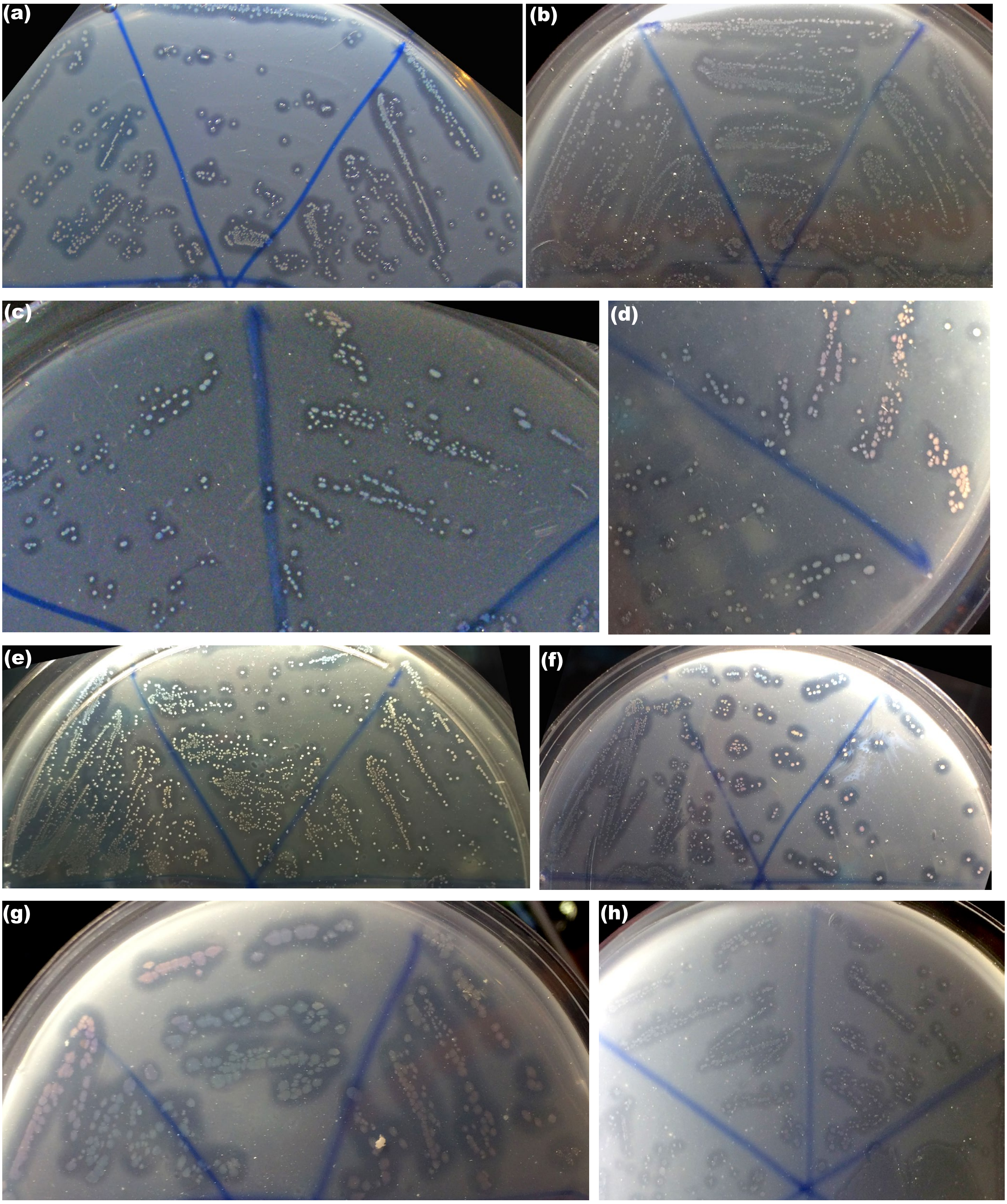

Fig.S1

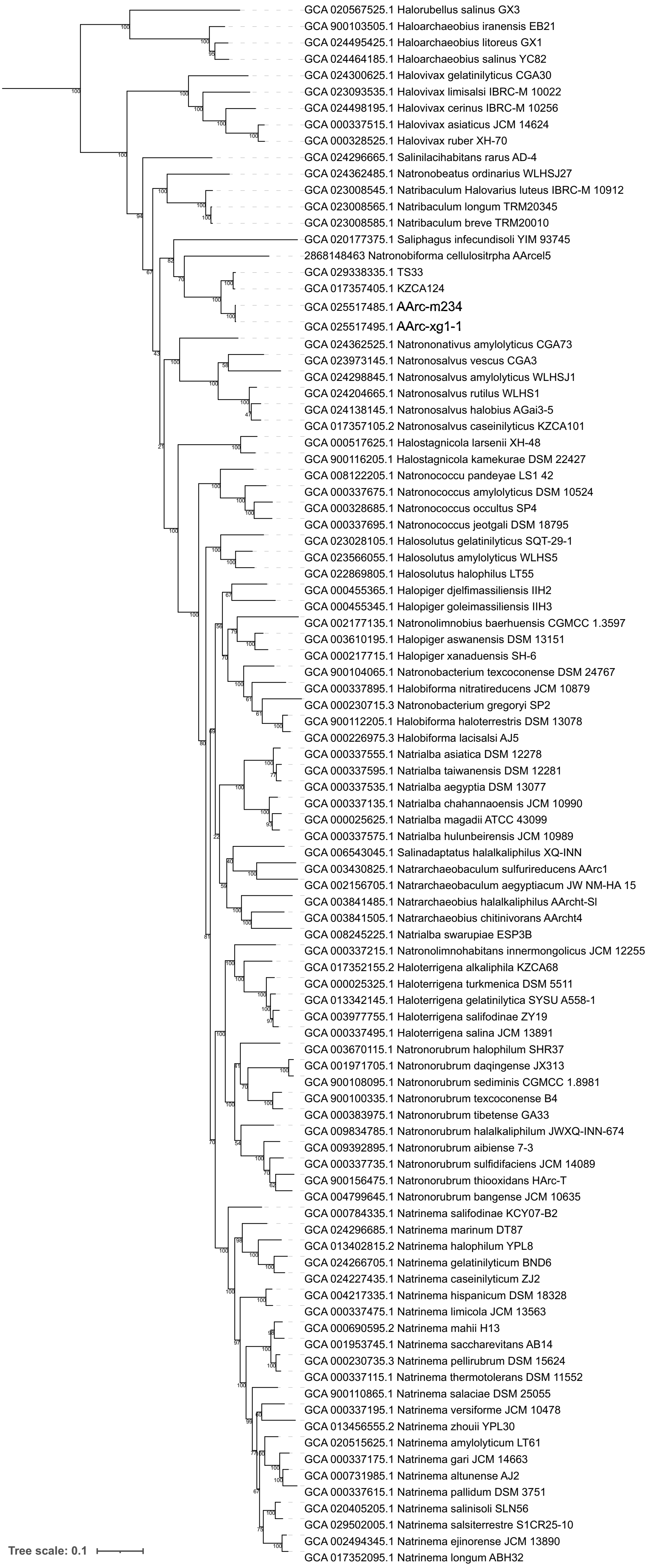
GCA 020567525.1 Halorubellus salinus GX3
GCA 900103505.1 Haloarchaeobius iranensis EB21
GCA 024495425.1 Haloarchaeobius litoreus GX1
GCA 024464185.1 Haloarchaeobius salinus YC82
GCA 024300625.1 Halovivax gelatinilyticus CGA30
GCA 023093535.1 Halovivax limisalsi IBRC-M 10022
GCA 024498195.1 Halovivax cerinus IBRC-M 10256
GCA 000337515.1 Halovivax asiaticus JCM 14624
GCA 000328525.1 Halovivax ruber XH-70
GCA 024296665.1 Salinilacihabitans rarus AD-4
GCA 024362485.1 Natronobeatus ordinarius WLHSJ27
GCA 023008545.1 Natribaculum Halovarius luteus IBRC-M 10912
GCA 023008565.1 Natribaculum longum TRM20345
GCA 023008585.1 Natribaculum breve TRM20010
GCA 020177375.1 Saliphagus infecundisoli YIM 93745
2868148463 Natronobiforma cellulositrpha AArcel5
GCA 029338335.1 TS33
GCA 017357405.1 KZCA124
GCA 025517485.1 AArc-m234
GCA 025517495.1 AArc-xg1-1
GCA 024362525.1 Natrononativus amylolyticus CGA73
GCA 023973145.1 Natronosalvus vescus CGA3
GCA 024298845.1 Natronosalvus amylolyticus WLHSJ1
GCA 024204665.1 Natronosalvus rutilus WLHS1
GCA 024138145.1 Natronosalvus halobius AGai3-5
GCA 017357105.2 Natronosalvus caseinilyticus KZCA101
GCA 000517625.1 Halostagnicola larsenii XH-48
GCA 900116205.1 Halostagnicola kamekurae DSM 22427
GCA 008122205.1 Natronococcu pandeyae LS1 42
GCA 000337675.1 Natronococcus amylolyticus DSM 10524
GCA 000328685.1 Natronococcus occultus SP4
GCA 000337695.1 Natronococcus jeotgali DSM 18795
GCA 023028105.1 Halosolutus gelatinilyticus SQT-29-1
GCA 023566055.1 Halosolutus amylolyticus WLHS5
GCA 022869805.1 Halosolutus halophilus LT55
GCA 000455365.1 Halopiger djelfimassiliensis IIH2
GCA 000455345.1 Halopiger goleimassiliensis IIH3
GCA 002177135.1 Natronolimnobius baerhuensis CGMCC 1.3597
GCA 003610195.1 Halopiger aswanensis DSM 13151
GCA 000217715.1 Halopiger xanaduensis SH-6
GCA 900104065.1 Natronobacterium texcoconense DSM 24767
GCA 000337895.1 Halobiforma nitratireducens JCM 10879
GCA 000230715.3 Natronobacterium gregoryi SP2
GCA 900112205.1 Halobiforma haloterrestris DSM 13078
GCA 000226975.3 Halobiforma lacisalsi AJ5
GCA 000337555.1 Natrialba asiatica DSM 12278
GCA 000337595.1 Natrialba taiwanensis DSM 12281
GCA 000337535.1 Natrialba aegyptia DSM 13077
GCA 000337135.1 Natrialba chahannaoensis JCM 10990
GCA 000025625.1 Natrialba magadii ATCC 43099
GCA 000337575.1 Natrialba hulunbeirensis JCM 10989
GCA 006543045.1 Salinadaptatus halalkaliphilus XQ-INN
GCA 003430825.1 Natrarchaeobaculum sulfurireducens AArc1
GCA 002156705.1 Natrarchaeobaculum aegyptiacum JW NM-HA 15
GCA 003841485.1 Natrarchaeobius halalkaliphilus AArcht-Sl
GCA 003841505.1 Natrarchaeobius chitinivorans AArcht4
GCA 008245225.1 Natrialba swarupiae ESP3B
GCA 000337215.1 Natronolimnohabitans innermongolicus JCM 12255
GCA 017352155.2 Haloterrigena alkaliphila KZCA68
GCA 000025325.1 Haloterrigena turkmenica DSM 5511
GCA 013342145.1 Haloterrigena gelatinilytica SYSU A558-1
GCA 003977755.1 Haloterrigena salifodinae ZY19
GCA 000337495.1 Haloterrigena salina JCM 13891
GCA 003670115.1 Natronorubrum halophilum SHR37
GCA 001971705.1 Natronorubrum daqingense JX313
GCA 900108095.1 Natronorubrum sediminis CGMCC 1.8981
GCA 900100335.1 Natronorubrum texcoconense B4
GCA 000383975.1 Natronorubrum tibetense GA33
GCA 009834785.1 Natronorubrum halalkaliphilum JWXQ-INN-674
GCA 009392895.1 Natronorubrum aibiense 7-3
GCA 000337735.1 Natronorubrum sulfidifaciens JCM 14089
GCA 900156475.1 Natronorubrum thiooxidans HArc-T
GCA 004799645.1 Natronorubrum bangense JCM 10635
GCA 000784335.1 Natrinema salifodinae KCY07-B2
GCA 024296685.1 Natrinema marinum DT87
GCA 013402815.2 Natrinema halophilum YPL8
GCA 024266705.1 Natrinema gelatinilyticum BND6
GCA 024227435.1 Natrinema caseinilyticum ZJ2
GCA 004217335.1 Natrinema hispanicum DSM 18328
GCA 000337475.1 Natrinema limicola JCM 13563
GCA 000690595.2 Natrinema mahii H13
GCA 001953745.1 Natrinema saccharevitans AB14
GCA 000230735.3 Natrinema pellirubrum DSM 15624
GCA 000337115.1 Natrinema thermotolerans DSM 11552
GCA 900110865.1 Natrinema salaciae DSM 25055
GCA 000337195.1 Natrinema versiforme JCM 10478
GCA 013456555.2 Natrinema zhouii YPL30
GCA 020515625.1 Natrinema amylolyticum LT61
GCA 000337175.1 Natrinema gari JCM 14663
GCA 000731985.1 Natrinema altunense AJ2
GCA 000337615.1 Natrinema pallidum DSM 3751
GCA 020405205.1 Natrinema salinisoli SLN56
GCA 029502005.1 Natrinema salsiterrestre S1CR25-10
GCA 002494345.1 Natrinema ejinorense JCM 13890
GCA 017352095.1 Natrinema longum ABH32
Tree scale: 0.1

Figure S2

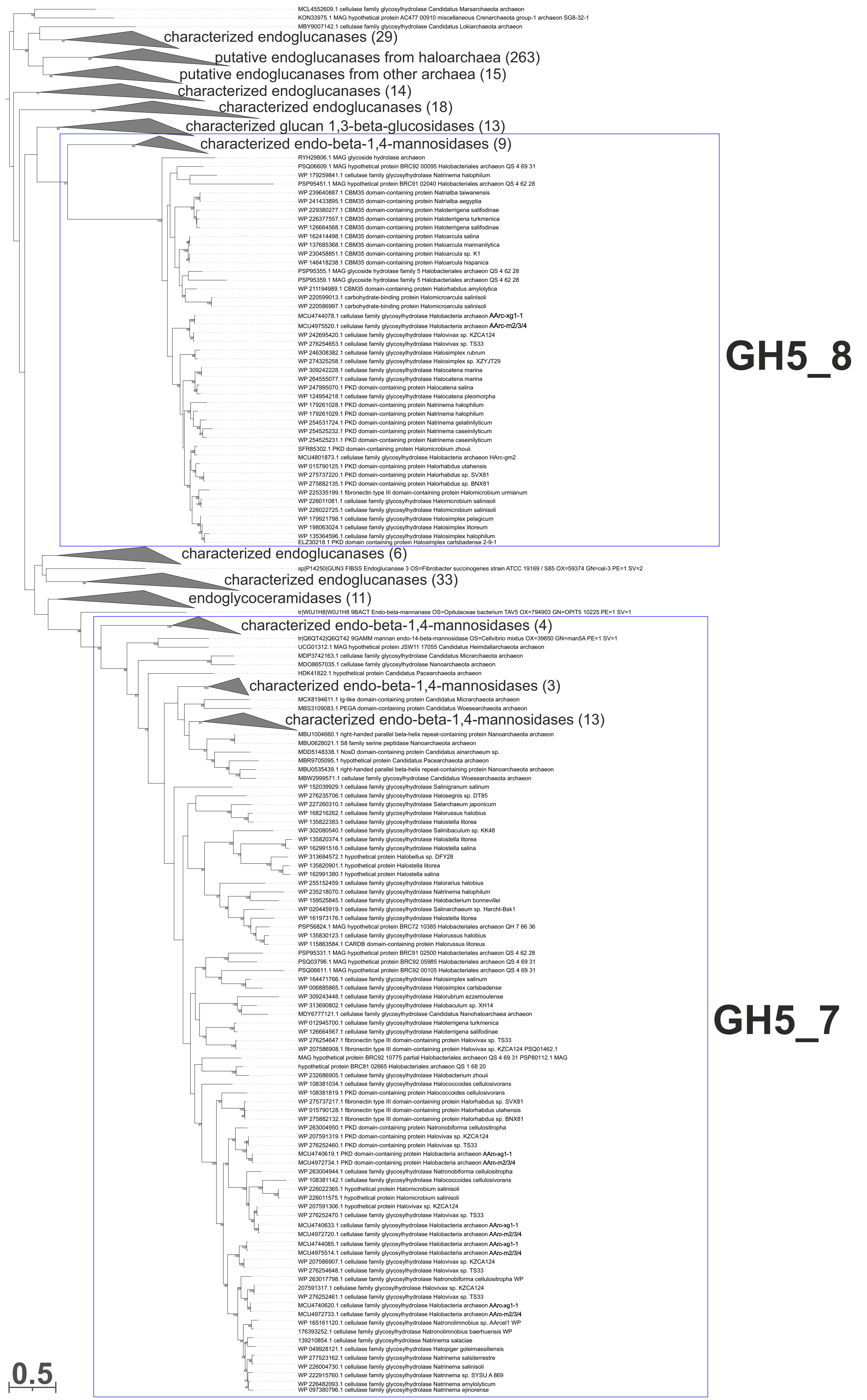
MCL4552609.1 cellulase family glycosylhydrolase Candidatus Marsarchaeota archaeon
KON33975.1 MAG hypothetical protein AC477 00910 miscellaneous Crenarchaeota group-1 archaeon SG8-32-1
MBY9007142.1 cellulase family glycosylhydrolase Candidatus Lokiarchaeota archaeon
characterized endoglucanases (29)
putative endoglucanases from haloarchaea (263)
putative endoglucanases from other archaea (15)
characterized endoglucanases (14)
characterized endoglucanases (18)
characterized glucan 1,3-beta-glucosidases (13)
characterized endo-beta-1,4-mannosidases (9)
RYH29806.1 MAG glycoside hydrolase archaeon
PSQ06609.1 MAG hypothetical protein BRC92 00095 Halobacteriales archaeon QS 4 69 31
WP 179259841.1 cellulase family glycosylhydrolase Natrinema halophilum
PSP95451.1 MAG hypothetical protein BRC91 02040 Halobacteriales archaeon QS 4 62 28
WP 239640887.1 CBM35 domain-containing protein Natrialba taiwanensis
WP 241433895.1 CBM35 domain-containing protein Natrialba aegyptia
WP 229380277.1 CBM35 domain-containing protein Haloterrigena salifodinae
WP 226377557.1 CBM35 domain-containing protein Haloterrigena turkmenica
WP 126664568.1 CBM35 domain-containing protein Haloterrigena salifodinae
WP 162414498.1 CBM35 domain-containing protein Haloarcula salina
WP 137685368.1 CBM35 domain-containing protein Haloarcula mannanilytica
WP 230458851.1 CBM35 domain-containing protein Haloarcula sp. K1
WP 146418238.1 CBM35 domain-containing protein Haloarcula hispanica
PSP95355.1 MAG glycoside hydrolase family 5 Halobacteriales archaeon QS 4 62 28
PSP95359.1 MAG glycoside hydrolase family 5 Halobacteriales archaeon QS 4 62 28
WP 211194989.1 CBM35 domain-containing protein Halorhabdus amylolytica
WP 220599013.1 carbohydrate-binding protein Halomicroarcula salinisoli
WP 220586997.1 carbohydrate-binding protein Halomicroarcula salinisoli
MCU4744078.1 cellulase family glycosylhydrolase Halobacteria archaeon AArc-xg1-1
MCU4975520.1 cellulase family glycosylhydrolase Halobacteria archaeon AArc-m2/3/4
WP 242695420.1 cellulase family glycosylhydrolase Halovivax sp. KZCA124
WP 276254653.1 cellulase family glycosylhydrolase Halovivax sp. TS33
WP 246308382.1 cellulase family glycosylhydrolase Halosimplex rubrum
WP 274325258.1 cellulase family glycosylhydrolase Halosimplex sp. XZYJT29
WP 309242228.1 cellulase family glycosylhydrolase Halocatena marina
WP 264555077.1 cellulase family glycosylhydrolase Halocatena marina
WP 247995070.1 PKD domain-containing protein Halocatena salina
WP 124954218.1 cellulase family glycosylhydrolase Halocatena pleomorpha
WP 179261028.1 PKD domain-containing protein Natrinema halophilum
WP 179261029.1 PKD domain-containing protein Natrinema halophilum
WP 254531724.1 PKD domain-containing protein Natrinema gelatinilyticum
WP 254525232.1 PKD domain-containing protein Natrinema caseinilyticum
WP 254525231.1 PKD domain-containing protein Natrinema caseinilyticum
SFR85302.1 PKD domain-containing protein Halomicrobium zhouii
MCU4801873.1 cellulase family glycosylhydrolase Halobacteria archaeon HArc-gm2
WP 015790125.1 PKD domain-containing protein Halorhabdus utahensis
WP 275737220.1 PKD domain-containing protein Halorhabdus sp. SVX81
WP 275882135.1 PKD domain-containing protein Halorhabdus sp. BNX81
WP 225335199.1 fibronectin type III domain-containing protein Halomicrobium urmianum
WP 226011081.1 cellulase family glycosylhydrolase Halomicrobium salinisoli
WP 226022725.1 cellulase family glycosylhydrolase Halomicrobium salinisoli
WP 179921798.1 cellulase family glycosylhydrolase Halosimplex pelagicum
WP 198063024.1 cellulase family glycosylhydrolase Halosimplex litoreum
WP 135364596.1 cellulase family glycosylhydrolase Halosimplex halophilum
ELZ30218.1 PKD domain containing protein Halosimplex carlsbadense 2-9-1
GH5_8
characterized endoglucanases (6)
sp|P14250|GUN3 FIBSS Endoglucanase 3 OS=Fibrobacter succinogenes strain ATCC 19169 / S85 OX=59374 GN=cel-3 PE=1 SV=2
characterized endoglucanases (33)
endoglycoceramidases (11)
tr|W0J1H8|W0J1H8 9BACT Endo-beta-mannanase OS=Opitutaceae bacterium TAV5 OX=794903 GN=OPIT5 10225 PE=1 SV=1
characterized endo-beta-1,4-mannosidases (4)
tr|Q6QT42|Q6QT42 9GAMM mannan endo-14-beta-mannosidase OS=Cellvibrio mixtus OX=39650 GN=man5A PE=1 SV=1
UCG01312.1 MAG hypothetical protein JSW11 17055 Candidatus Heimdallarchaeota archaeon
MDP3742163.1 cellulase family glycosylhydrolase Candidatus Micrarchaeota archaeon
MDO8657035.1 cellulase family glycosylhydrolase Nanoarchaeota archaeon
HDK41822.1 hypothetical protein Candidatus Pacearchaeota archaeon
characterized endo-beta-1,4-mannosidases (3)
MCX8194611.1 Ig-like domain-containing protein Candidatus Micrarchaeota archaeon
MBS3109083.1 PEGA domain-containing protein Candidatus Woesearchaeota archaeon
characterized endo-beta-1,4-mannosidases (13)
MBU1004660.1 right-handed parallel beta-helix repeat-containing protein Nanoarchaeota archaeon
MBU0628021.1 S8 family serine peptidase Nanoarchaeota archaeon
MDD5148338.1 NosD domain-containing protein Candidatus ainarchaeum sp.
MBR9705095.1 hypothetical protein Candidatus Pacearchaeota archaeon
MBU0535439.1 right-handed parallel beta-helix repeat-containing protein Nanoarchaeota archaeon
MBW2999571.1 cellulase family glycosylhydrolase Candidatus Woesearchaeota archaeon
WP 152039929.1 cellulase family glycosylhydrolase Saliniigranum salinum
WP 276235706.1 cellulase family glycosylhydrolase Halosegnis sp. DT85
WP 227260310.1 cellulase family glycosylhydrolase Salarchaeum japonicum
WP 168216262.1 cellulase family glycosylhydrolase Halorussus halobius
WP 135822383.1 cellulase family glycosylhydrolase Halostella litorea
WP 302080540.1 cellulase family glycosylhydrolase Salinibaculum sp. KK48
WP 135820374.1 cellulase family glycosylhydrolase Halostella litorea
WP 162991516.1 cellulase family glycosylhydrolase Halostella salina
WP 313684572.1 hypothetical protein Halobellus sp. DFY28
WP 135820901.1 hypothetical protein Halostella litorea
WP 162991380.1 hypothetical protein Halostella salina
WP 255152459.1 cellulase family glycosylhydrolase Halorarius halobius
WP 235218070.1 cellulase family glycosylhydrolase Natrinema halophilum
WP 159525845.1 cellulase family glycosylhydrolase Halobacterium bonnevillei
WP 020445919.1 cellulase family glycosylhydrolase Salinarchaeum sp. Harcht-Bsk1
WP 161973176.1 cellulase family glycosylhydrolase Halostella litorea
PSP56824.1 MAG hypothetical protein BRC72 10385 Halobacteriales archaeon QH 7 66 36
WP 135830123.1 cellulase family glycosylhydrolase Halorussus halobius
WP 115863584.1 CARDB domain-containing protein Halorussus litoreus
PSP95331.1 MAG hypothetical protein BRC91 02500 Halobacteriales archaeon QS 4 62 28
PSQ03796.1 MAG hypothetical protein BRC92 05985 Halobacteriales archaeon QS 4 69 31
PSQ06611.1 MAG hypothetical protein BRC92 00105 Halobacteriales archaeon QS 4 69 31
WP 164471766.1 cellulase family glycosylhydrolase Halosimplex salinum
WP 006885865.1 cellulase family glycosylhydrolase Halosimplex carlsbadense
WP 309243448.1 cellulase family glycosylhydrolase Halorubrum ezzemoulense
WP 313690802.1 cellulase family glycosylhydrolase Halobaculum sp. XH14
MDY6777121.1 cellulase family glycosylhydrolase Candidatus Nanohaloarchaea archaeon
WP 012945700.1 cellulase family glycosylhydrolase Haloterrigena turkmenica
WP 126664567.1 cellulase family glycosylhydrolase Haloterrigena salifodinae
WP 276254647.1 fibronectin type III domain-containing protein Halovivax sp. TS33
WP 207586908.1 fibronectin type III domain-containing protein Halovivax sp. KZCA124 PSQ01462.1
MAG hypothetical protein BRC92 10775 partial Halobacteriales archaeon QS 4 69 31 PSP80112.1 MAG
hypothetical protein BRC81 02665 Halobacteriales archaeon QS 1 68 20
WP 232686905.1 cellulase family glycosylhydrolase Halobacterium zhouii
WP 108381034.1 cellulase family glycosylhydrolase Halococcoides cellulosivorans
WP 108381819.1 PKD domain-containing protein Halococcoides cellulosivorans
WP 275737217.1 fibronectin type III domain-containing protein Halorhabdus sp. SVX81
WP 015790128.1 fibronectin type III domain-containing protein Halorhabdus utahensis
WP 275882132.1 fibronectin type III domain-containing protein Halorhabdus sp. BNX81
WP 263004950.1 PKD domain-containing protein Natronobiforma cellulositropha
WP 207591319.1 PKD domain-containing protein Halovivax sp. KZCA124
WP 276252460.1 PKD domain-containing protein Halovivax sp. TS33
MCU4740619.1 PKD domain-containing protein Halobacteria archaeon AArc-xg1-1
MCU4972734.1 PKD domain-containing protein Halobacteria archaeon AArc-m2/3/4
WP 263004944.1 cellulase family glycosylhydrolase Natronobiforma cellulositropha
WP 108381142.1 cellulase family glycosylhydrolase Halococcoides cellulosivorans
WP 226022365.1 hypothetical protein Halomicrobium salinisoli
WP 226011575.1 hypothetical protein Halomicrobium salinisoli
WP 207591306.1 hypothetical protein Halovivax sp. KZCA124
WP 276252470.1 cellulase family glycosylhydrolase Halovivax sp. TS33
MCU4740633.1 cellulase family glycosylhydrolase Halobacteria archaeon AArc-xg1-1
MCU4972720.1 cellulase family glycosylhydrolase Halobacteria archaeon AArc-m2/3/4
MCU4744085.1 cellulase family glycosylhydrolase Halobacteria archaeon AArc-xg1-1
MCU4975514.1 cellulase family glycosylhydrolase Halobacteria archaeon AArc-m2/3/4
WP 207586907.1 cellulase family glycosylhydrolase Halovivax sp. KZCA124
WP 276254648.1 cellulase family glycosylhydrolase Halovivax sp. TS33
WP 263017798.1 cellulase family glycosylhydrolase Natronobiforma cellulositropha WP
207591317.1 cellulase family glycosylhydrolase Halovivax sp. KZCA124
WP 276252461.1 cellulase family glycosylhydrolase Halovivax sp. TS33
MCU4740620.1 cellulase family glycosylhydrolase Halobacteria archaeon AArc-xg1-1
MCU4972733.1 cellulase family glycosylhydrolase Halobacteria archaeon AArc-m2/3/4
WP 165161120.1 cellulase family glycosylhydrolase Natronolimnobius sp. AArcel1 WP
176393252.1 cellulase family glycosylhydrolase Natronolimnobius baerhuensis WP
139210854.1 cellulase family glycosylhydrolase Natrinema salaciae
WP 049928121.1 cellulase family glycosylhydrolase Halopiger goleimassiliensis
WP 277523162.1 cellulase family glycosylhydrolase Natrinema salsiterrestre
WP 226004730.1 cellulase family glycosylhydrolase Natrinema salinisoli
WP 222915760.1 cellulase family glycosylhydrolase Natrinema sp. SYSU A 869
WP 226482093.1 cellulase family glycosylhydrolase Natrinema amylolyticum
WP 097380796.1 cellulase family glycosylhydrolase Natrinema ejinorense
GH5_7
0.5

Figure S3
